# Label-Free In Situ Chemical Characterization of Amyloid Plaques in Human Brain Tissues

**DOI:** 10.1021/acschemneuro.3c00756

**Published:** 2024-03-19

**Authors:** James Everett, Jake Brooks, Vindy Tjendana Tjhin, Frederik Lermyte, Ian Hands-Portman, Germán Plascencia-Villa, George Perry, Peter J. Sadler, Peter B. O’Connor, Joanna F. Collingwood, Neil D. Telling

**Affiliations:** † School of Pharmacy and Bioengineering, Guy Hilton Research Centre, Keele University, Thornburrow Drive,Stoke-on-Trent,Staffordshire ST4 7QB, U.K.; ‡ School of Engineering, University of Warwick, Library Road,Coventry CV4 7AL, U.K.; § Department of Chemistry, Technical University of Darmstadt, Alarich-Weiss-Strasse 4, 64287 Darmstadt, Germany; ∥ School of Life Sciences, University of Warwick, Gibbet Hill Campus,Coventry CV4 7AL, U.K.; ⊥ Department of Developmental and Regenerative Biology, The University of Texas at San Antonio (UTSA), San Antonio, Texas 78249, United States; # Department of Chemistry, University of Warwick, Library Road,Coventry CV4 7AL, U.K.

**Keywords:** Alzheimer’s disease, amyloid plaques, X-ray spectromicroscopy, STXM, metals

## Abstract

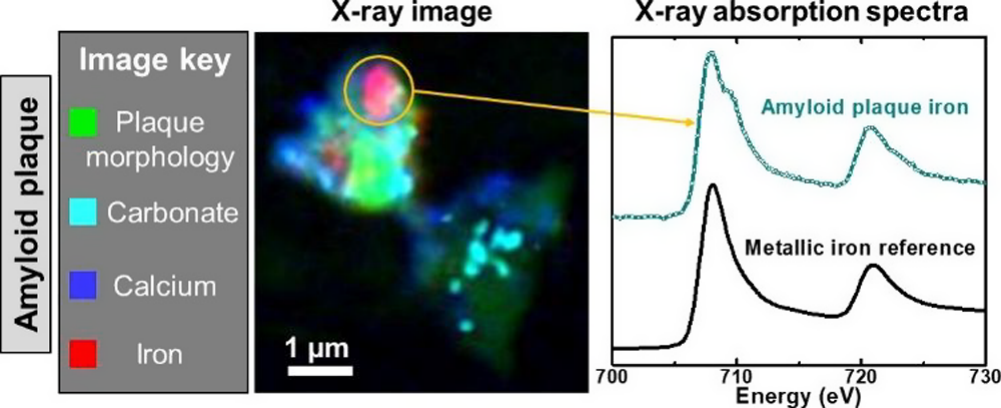

The accumulation of amyloid plaques and increased brain redox burdens are neuropathological hallmarks of Alzheimer’s disease. Altered metabolism of essential biometals is another feature of Alzheimer’s, with amyloid plaques representing sites of disturbed metal homeostasis. Despite these observations, metal-targeting disease treatments have not been therapeutically effective to date. A better understanding of amyloid plaque composition and the role of the metals associated with them is critical. To establish this knowledge, the ability to resolve chemical variations at nanometer length scales relevant to biology is essential. Here, we present a methodology for the label-free, nanoscale chemical characterization of amyloid plaques within human Alzheimer’s disease tissue using synchrotron X-ray spectromicroscopy. Our approach exploits a C–H carbon absorption feature, consistent with the presence of lipids, to visualize amyloid plaques selectively against the tissue background, allowing chemical analysis to be performed without the addition of amyloid dyes that alter the native sample chemistry. Using this approach, we show that amyloid plaques contain elevated levels of calcium, carbonates, and iron compared to the surrounding brain tissue. Chemical analysis of iron within plaques revealed the presence of chemically reduced, low-oxidation-state phases, including ferromagnetic metallic iron. The zero-oxidation state of ferromagnetic iron determines its high chemical reactivity and so may contribute to the redox burden in the Alzheimer’s brain and thus drive neurodegeneration. Ferromagnetic metallic iron has no established physiological function in the brain and may represent a target for therapies designed to lower redox burdens in Alzheimer’s disease. Additionally, ferromagnetic metallic iron has magnetic properties that are distinct from the iron oxide forms predominant in tissue, which might be exploitable for the in vivo detection of amyloid pathologies using magnetically sensitive imaging. We anticipate that this label-free X-ray imaging approach will provide further insights into the chemical composition of amyloid plaques, facilitating better understanding of how plaques influence the course of Alzheimer’s disease.

## Introduction

Alzheimer’s disease (AD) is a fatal progressive neurodegenerative disorder and the leading cause of dementia worldwide.^1^ Clinically, AD presents as progressive memory loss, psychosis, and cognitive decline.^2−4^ The primary risk factor for AD is age, with disease onset most common in people over the age of 65.^5,6^ The underlying pathogenesis of AD is highly complex and is yet to be fully understood. As a result, no cure or effective disease-modifying treatment currently exists, only treatments to control symptomology.^7^ With average life expectancies set to rise in the coming decades, worldwide AD cases are projected to triple by 2050,^5^ representing an impending crisis of burden on healthcare and societal infrastructure globally. As such, the World Health Organization has declared the need for improved strategies for AD disease prevention and treatment as a top research priority.

Pathophysiologically, the AD brain is characterized by the presence of two hallmark protein lesions: (i) intracellular neurofibrillary tangles (NFTs) composed of aggregates of hyperphosphorylated tau protein^6,8,9^ and (ii) extracellular amyloid plaques, primarily composed of the amyloid-β peptide.^6,10−12^ Although some debate remains over whether these subcellular-sized pathologies are a cause of AD or an effect of unresolved upstream cellular mechanisms, their accumulation and association with disrupted brain functions (neuronal and synaptic failure and neuroinflammation) are recognized as key events in AD.

Disrupted brain metal homeostasis has been observed in multiple neurodegenerative disorders including AD, Parkinson’s, Huntington’s, Wilson’s, and amyotrophic lateral sclerosis,^13−20^ linking erroneous metal handling by the aging brain and the onset of neurodegeneration. The human central nervous system utilizes a variety of metals for many essential processes, with sophisticated regulatory systems ensuring uptake and trafficking of bioavailable metal ions.^17,21,22^ Although critical to brain function, metals can convey toxic effects when stored incorrectly or when abnormal chemical states are adopted. For example, low molecular-weight, weakly bound labile iron and copper can partake in redox chemistry, resulting in the overproduction of harmful reactive oxygen species (ROS) capable of damaging cellular components (lipid membranes, proteins, and DNA/RNA), ultimately leading to neuronal failure and cell death.^23,24^ Low-oxidation-state metals can catalyze these reactions, further driving ROS overproduction. In AD, amyloid plaques and neurofibrillary tangles are highly active sites of impaired metal homeostasis.^12,25−30^ An example of this is colocalization of low-oxidation-state (i.e., < +3) and magnetic iron in amyloid plaques,^31−33^ giving rise to the possibility that their occurrence might be connected with the development of lesions in the presence of endogenously or exogenously acquired metals.

Despite this evidence, the chemistry of metal/protein interactions and the diverse metal chemistry within AD pathology are poorly understood. Metal chemistry in biological systems can vary dramatically over small (<100 nm) spatial scales.^28,34^ Therefore, to understand these complex systems, imaging and chemical sensitivity with nanoscale resolution are essential. However, the current knowledge of metal biochemistry in neurodegenerative diseases lacks this level of detail. Further, traditional histological techniques used to examine neuropathology and (metal) biochemistry in the AD brain rely on the use of chemical dyes, contrast agents, and aldehyde fixatives, all of which can significantly alter the native chemistry of the sample material being examined.^35^ The use of these approaches hinders our understanding of protein lesions and metal biochemistry in the AD brain and how these factors together may contribute to disease pathogenesis.

The lack of detailed understanding of the underlying metal chemistry of AD has arguably contributed to the limited efficacy of metal-targeting pharmacological strategies to date.^36,37^ A reason for these shortcomings is the inability of therapies to distinguish between the metal species required for healthy brain functions and those associated with diseased states. Thus, it is of paramount importance to characterize metal phases uniquely associated with disease states and distinguish them from those involved in normal metabolism in the human brain. Furthermore, the precise biochemical composition of amyloid plaques and neurofibrillary tangles remains undefined, limiting our understanding of how these pathologies contribute to disease development and hindering the efficacy of plaque and tangle targeting therapies. There is an urgent need to address these outstanding questions and improve the disease prognosis.

One novel technique that offers chemically sensitive nanoscale resolution imaging is scanning transmission X-ray microscopy (STXM), a form of X-ray spectromicroscopy.^38^ STXM allows the simultaneous collection of both chemically sensitive images and detailed X-ray absorption spectra to a spatial resolution of <50 nm, revealing the distribution of different biochemical constituents within a sample. This approach enables sample composition to be determined as a function of two-dimensional space, with a level of spatial and chemical precision inaccessible using techniques traditionally aligned to the biological sciences.

The large accessible energy range of STXM allows the imaging of both organic and inorganic X-ray absorption edges and correlations between metal chemistry and specific biological structures (e.g., protein deposits) to be identified simultaneously.^38^ Importantly, STXM does not require the use of any dyes, aldehyde fixatives, or contrast agents used in conventional imaging techniques, while careful regulation of X-ray photon doses ensures that the biochemistry of the sample is not disturbed upon measurement. Additionally, through the use of circularly polarized X-rays, the magnetic properties of the material in a given region of interest (ROI) can be correlated to the chemical speciation of that same ROI via the X-ray magnetic circular dichroism (XMCD) effect.^39^

In previous work using STXM, we reported the ground-breaking discovery of elemental metallic copper and iron nanodeposits in amyloid plaque cores isolated from AD tissue.^28,34^ Iron and copper in these highly reactive forms, previously undocumented in the human tissue, are capable of generating free radicals which could contribute to the pattern of redox stress and cell loss observed in AD.^13,36,37^ Furthermore, metallic iron is strongly (ferro)magnetic and so can be visualized using magnetically sensitive imaging techniques.^40^ Continued advancements in magnetic resonance imaging (MRI)^41^ may support future exploitation of the magnetic properties of ferromagnetic iron for the imaging of amyloid pathologies in a clinical setting. These MRI advances include increased access to higher spatial resolution using clinical instruments and refinement of methods (such as quantitative susceptibility mapping and transverse relaxation imaging) that are complementary in their relative sensitivities to magnetic properties and other tissue property changes associated with neurodegeneration.^42−44^ Thus, metallic nanoparticles occurring within the human brain offer potential new targets for metal-targeting therapies for AD treatment and diagnosis. Our discovery of these nanoscale deposits in isolated AD plaque cores was made possible through the use of these state-of-the-art X-ray techniques.^28,34^

Here, we apply STXM techniques to human AD brain tissue sections to determine their chemical and mineral composition. We describe a methodology for the label-free in situ visualization of amyloid plaques at nanoscale resolution using an absorption feature characteristic of carbon–hydrogen bonds (C–H) that is prominent in the plaques but not in the surrounding tissue parenchyma. This approach allows the discrimination between amyloid plaques and the tissue background, enabling the chemical composition of amyloid plaques to be explored without the addition of chemical dyes or contrast agents. We demonstrate that amyloid plaques contain elevated levels of calcium, carbonates, and iron when compared to the surrounding tissue. Chemical analysis of the plaque iron revealed the presence of ferromagnetic metallic iron, the first demonstration of this in intact tissue sections, and fully consistent with our reported observation of elemental metallic nanoparticles within isolated amyloid plaque cores.

It is anticipated that label-free X-ray imaging will further elucidate the chemical composition of amyloid plaques, advancing our understanding of the role of these lesions in AD pathogenesis.

## Results and Discussion

The goal of this work was to examine the biochemical composition of brain tissue sections from aged human subjects, with a focus on the chemical profiling of amyloid plaques, a brain lesion associated with the development of neurodegenerative disorders including AD. STXM was the chosen method to examine the brain tissue owing to its combined chemical and spatial sensitivity, allowing in-depth chemical characterization of the brain tissue and amyloid plaques contained within. These approaches allowed new insights into the role played by amyloid pathologies in the biology of AD to be realized.

### STXM Visualization of the Brain Tissue Structure

To assess the brain tissue preservation following resin embedding and sectioning, 500 nm thick sections of putamen and 200 nm thick sections of amygdala and hippocampal tissue were examined using STXM at the oxygen and carbon *K*-edges.

**Figure 1:**
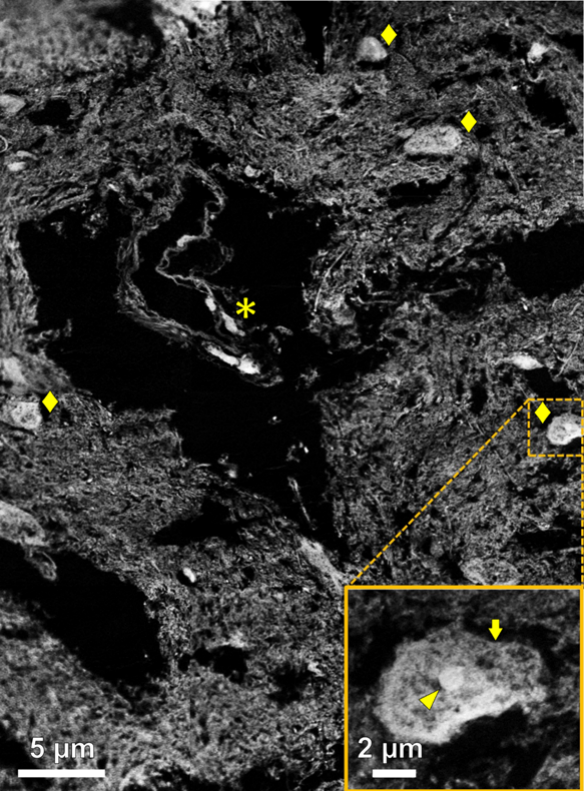
Oxygen *K*-edge tissue speciation maps from a 500 nm thick AD putamen tissue section. Mapping was performed at 532 eV to a spatial resolution of 100 nm. ◆ and _*_ symbols in the main image highlight cellular bodies and suspected blood vessel, respectively. The inset shows a zoomed image of the area highlighted by the dashed yellow box, containing a cellular body where the subcellular cell membrane (arrow) and nucleolus (arrowhead) are apparent.

To visualize tissue preservation in 500 nm thick sections, speciation maps were collected at 532 eV, the energy of the O 1s → π* transition for COO/COOH/CONH_2_ groups.^45^ The resulting tissue maps, an example of which is shown in Figure 1, revealed identifiable cellular bodies, blood vessels, and extracellular neuropil components of the brain tissue. Cell bodies typically appeared dense against the neuropil background and displayed recognizable subcellular features such as the cell membrane and cell nucleus/nucleolus (Figure 1 inset). These findings demonstrated the following: (i) satisfactory preservation of tissue ultrastructure during the resin embedding and sectioning process. (ii) The high chemical sensitivity and spatial resolution of STXM to be sufficient to unequivocally identify organic tissue features on length scales consistent with protein-related lesions such as amyloid plaques.

**Figure 2:**
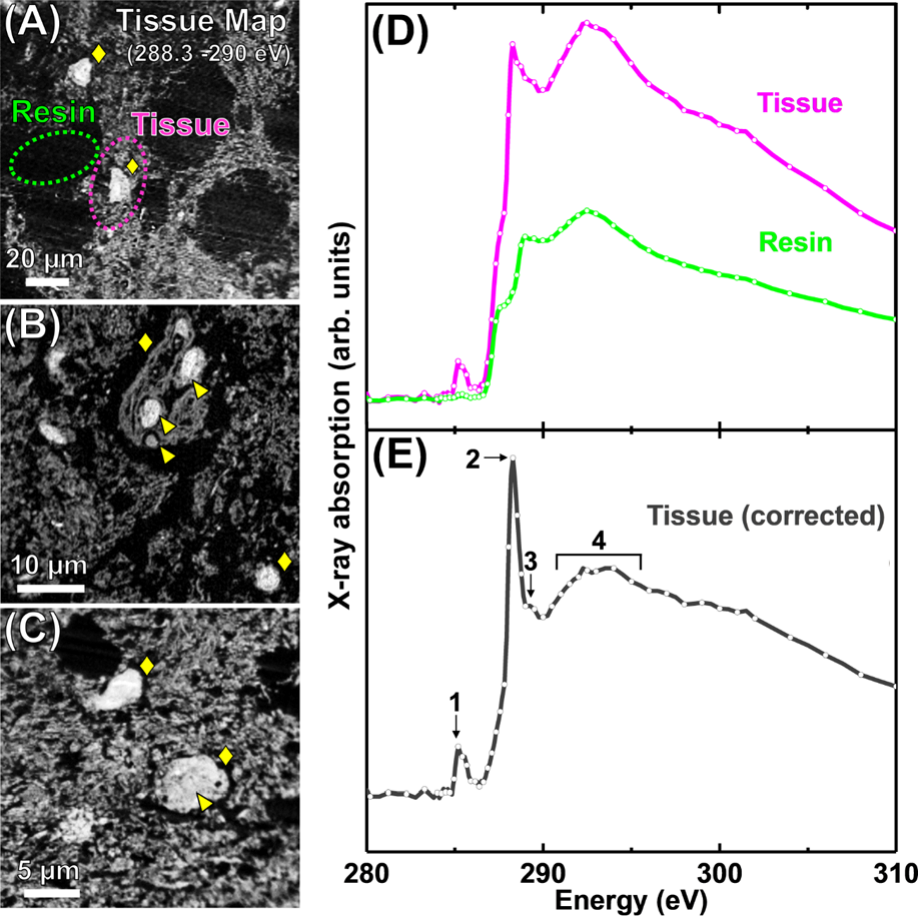
Carbon *K*-edge tissue speciation maps (A–C) and X-ray absorption spectra (D, E) from 200 nm thick AD amygdala (A, C–E) and hippocampal (B) tissue sections. (A–C) Tissue speciation maps performed at 288.3 eV to a spatial resolution of 150 nm. ◆ symbols highlight cellular bodies and arrowheads subcellular structures (e.g., nucleus/nucleolus in (C)). (D) X-ray absorption spectra from resin and tissue regions are highlighted in panel (A). (E) Tissue spectrum created through the subtraction of the resin spectrum (green trace) from the tissue spectrum (pink trace) in (D). Principal peak positions in the corrected tissue spectrum are labeled 1–4. Bond assignments for these peak positions are listed in Table 1.

In 200 nm thick sections of amygdala and hippocampal tissue, tissue preservation was assessed by performing speciation mapping at 288.3 eV, the energy of the C 1s → π* transition for the C=O bonds of amide groups.^46−48^ Consistent with measurements performed on the 500 nm sections, cellular bodies and subcellular components within were recognizable against the neuropil background in all different areas analyzed (Figure 2A–C). Larger areas of the section were devoid of the tissue structure (i.e., they were blank resin) in 200 nm thick sections compared to the 500 nm sections, as would be expected due to the lower volume of tissue in these thinner sections.

In addition to tissue speciation maps, representative carbon *K*-edge X-ray absorption spectra from the brain tissue parenchyma were collected by performing spectromicroscopy over the entire carbon *K*-edge. These spectra provide detailed information on the organic composition of the tissue and can be used to assess heterogeneity in sample composition across the section area.

Carbon *K*-edge X-ray absorption spectra acquired from the tissue section area in Figure 2A are shown in Figure 2D. The pink spectrum represents sample regions containing tissue, and the green spectrum is the background blank resin. As described in our previous work^49,50^ by subtracting the resin spectrum from the tissue spectrum, a corrected tissue spectrum can be achieved that is free of absorption artifacts from the embedding matrix, thereby revealing the chemical state of the tissue. The weighting of resin subtraction is determined by scaling the carbon *K*-edge resin spectrum to the pre-edge shoulder in the tissue spectrum and then subtracting the appropriately scaled resin spectrum. This corrected tissue spectrum, shown in Figure 2E, is composed of four principal absorption features (see also Table 1). Peak 1 is a low-intensity peak at ca. 285 eV, corresponding to C 1s → π* transition of C=C bonds,^51^ commonly attributed to the aromatic groups of the amino acids tyrosine, phenylalanine, and tryptophan in protein-based structures.^47^ This feature also corresponds to the C 1s → π* transition of C=C bonds from aliphatic alkene groups, such as those present in the aliphatic chains of unsaturated fatty acids.^51^ Peak 2 is a dominant, high-intensity white line peak at 288.3 eV, corresponding to the C 1s → π* transition for the C=O bond of the amide group found in peptide bonds.^46^ Peak 3 is a low-intensity feature at 289.3 eV associated with C 1s → π* transition of unsaturated C=N bonds from, e.g., arginine.^46,48^ Finally, peak 4 is a broad, shallow absorption feature spanning 292–295 eV, corresponding to the C 1s → σ* transition for C–C bonds.^52^ This spectrum was found to be consistent across multiple tissue parenchyma regions (for further examples, see Figure S1).

**Table 1:** Bond Assignments for the Absorption Features of the Corrected Tissue Spectrum Are Displayed in Figure 2E

peak number	energy (eV)	bond assignment (s)
1	∼285	1s → π* C=C aromatic; 1s → π* C=C alkene
2	288.3	1s → π* C=O amide
3	289.3	1s → π* C=N
4	292–295	1s → σ* C–C (broad)

### Carbon Spectromicroscopy of Isolated Amyloid Plaques

Following the confirmation of tissue structure preservation and the acquisition of a representative carbon *K*-edge absorption spectrum for the tissue parenchyma in the sections of amygdala and hippocampus; 200 nm thick sections containing isolated amyloid plaques from two confirmed (Braak stage VI) AD cases were examined at the carbon *K*-edge by STXM mapping. Congo red staining of additional sections cut from the same regions as those imaged by STXM confirmed the presence of amyloid plaques within the embedded material (see Figure 1 of Everett et al.^34^). By examining amyloid plaques in isolation, we sought to establish commonalities and differences in the carbon composition of the plaques when compared with the tissue parenchyma. Should the amyloid plaques provide absorption features that are not present in the tissue parenchyma, these features could be exploited for label-free in situ identification of amyloid plaques against the prevailing tissue background within brain tissue sections.

**Figure 3:**
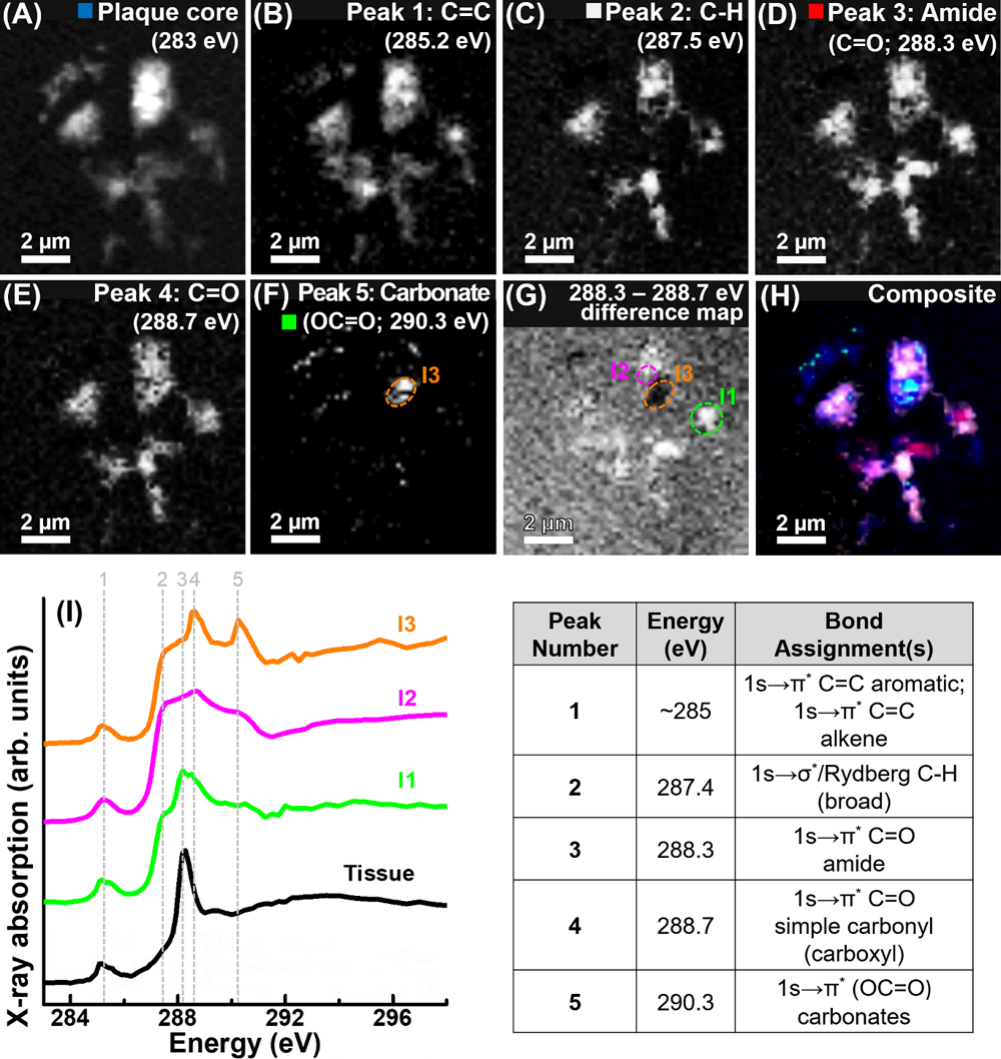
Carbon *K*-edge X-ray images speciation maps and X-ray absorption spectra of an isolated amyloid plaque core from subject X. (A) Single energy 283 eV image showing the overall plaque morphology. (B) C=C map, corresponding to peak 1 in (I). (C) C–H map, peak 2 in (I). (D) Amide map, peak 3 in (I). (E) C=O map, peak 4 in (I). (F) Carbonate map, peak 5 in (I). (G) Carbon chemistry difference map, where areas of light contrast strongly absorb at the C=O amide feature [288.3 eV; peak 3 in (I)] and areas of dark contrast strongly absorb at the C=O simple carbonyl feature [288.7 eV; peak 4 in (I)]. (H) Composite image showing the morphology (blue), C–H (gray), amide (red), and carbonate (green) content of the plaque. (I) Carbon *K*-edge X-ray absorption spectra (labeled I1–I3; colored traces) from the plaque areas highlighted in panels (F) and (G). A tissue parenchyma spectrum (from Figure 2E) is provided for comparison. Five principal absorption features are shown by dotted lines, with the associated table providing bond assignments for these five features.

Carbon *K*-edge X-ray images, speciation maps, and X-ray absorption spectra of an isolated amyloid plaque core are shown in Figure 3. Prior STXM analysis of the same plaque structure is shown in Figure 4a of Everett et al.^34^ The carbon *K*-edge X-ray spectromicroscopy data sets were used previously merely to confirm the presence of a biological (plaque) material within the embedding resin, focusing on establishing the metal biochemistry within amyloid plaque cores. Here, we examine and exploit the characteristic features of the carbon *K*-edge spectromicroscopy data sets from the amyloid plaques in greater detail.

Carbon *K*-edge X-ray absorption spectra from the amyloid plaque areas highlighted in Figure 3F,G are displayed in Figure 3I, alongside a tissue parenchyma reference spectrum (as shown in Figure 2E) for comparison. Bond assignments for the principal X-ray absorption features labeled in panel I are listed in Figure 3. Examination of the amyloid plaque carbon spectra (I1–I3) showed that the organic composition was heterogeneous, with localized differences in absorption features observed. Importantly, several features not present in the tissue parenchyma spectrum of Figure 2E were evident.

Spectra from all amyloid plaque regions displayed a C=C peak at 285 eV, as observed in the tissue reference spectrum (peak 1; panel B for the associated speciation map). However, all plaque regions also displayed a broad absorption feature at 287.5 eV (peak 2; panel C), corresponding to the C 1s → σ*/Rydberg transition for C–H groups,^47,52,53^ which was not discernible in the tissue reference spectrum. This feature has previously been identified in carbon *K*-edge absorption spectra from phospholipids,^54^ likely arising from the aliphatic hydrocarbon chains of fatty acids, suggesting similar groups to be present in the amyloid plaque cores. Indeed, lipids have previously been identified as organic components present around and within amyloid plaques in AD tissues.^55−58^

Importantly, the presence and intensity (relative to maximum carbon *K*-edge absorption) of the C–H 287.5 eV absorption feature in the plaques were not an artifact of X-ray beam saturation or sample density as this feature was (i) consistently observed in different plaque regions of varying optical density, including those considerably below the saturation limits; and (ii) its intensity was not consistently positively correlated with optical density (see Supporting Information Figure S2).

While the tissue spectrum included a dominant white line peak at 288.3 eV arising from the C=O group of amide bonds (peak 3; panel D), only spectrum I1 from the three plaque spectra in Figure 3 strongly displayed this absorption feature, albeit it was broadened and shallower than in the tissue reference. In plaque areas I2 and I3, the C=O peak feature is present at a higher energy, consistent with C 1s → π* transitions for C=O bonds in simple carbonyl groups (peak 4; panel E). As with the C–H absorption feature at 287.5 eV, the carbonyl feature at ca. 288.7 eV is present in a reference phospholipid spectrum^54^ but not the tissue reference from Figure 2E. The red shift (commonly called the peptide shift) in the C 1s → π* C=O peak position of proteins compared to, for example, saccharides is well documented and arises when adjacent carbon atoms in the C–C(=O)–C structure are replaced by nitrogen atoms, such as in the amide bonds of proteins/peptides.^46^ Taking into account these two C=O peak positions, the broad shape of the C=O peak in plaque spectrum I1 indicates the presence of both amide and simple carbonyl groups in this area. As described in Everett et al.,^59^ the plaques also contained carbonate deposits, as evidenced though the sharp C 1s → π* carbonate absorption peak at 290.3 eV in plaque spectrum I3 (peak 5; panel F).^60^

Carbon *K*-edge spectromicroscopy data from three further plaques including a second AD case are provided in Supporting Information Figures S3–S5 and similarly showed strong absorption at 287.5 eV when compared to the tissue reference, indicating that this feature can be used to visualize plaque structures against the tissue background within brain tissue sections.

### Label-Free STXM Analysis of Amyloid Plaques within AD Tissues

To assess whether the 287.5 eV feature observed in the carbon *K*-edge X-ray absorption spectra from isolated amyloid plaques could be used to visualize amyloid pathology within the sections of brain tissue in situ, STXM was performed on 200 nm thick sections of amygdala and hippocampus tissue from two AD cases with confirmed evidence of neuropathology (Braak stage VI).

**Figure 4:**
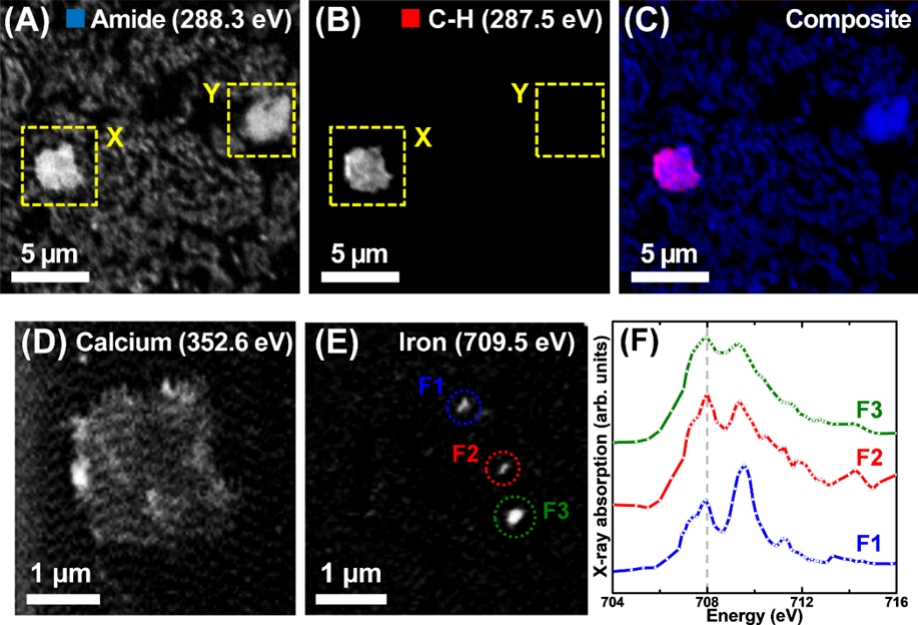
STXM examination of an amyloid plaque within an AD hippocampal tissue section. (A–C) Carbon *K-*edge mapping. (A) Amide map showing the overall tissue morphology. (B) C–H map revealing an amyloid plaque (labeled structure X). (C) Composite image showing amide (blue) and C–H (red) distribution. (D–F) Metal speciation maps and X-ray absorption spectra from the amyloid plaque (structure X in panels A, B). (D) Calcium *L*-edge map. (E) Iron *L*_3_-edge map. (F) Iron *L*_3_-edge X-ray absorption spectra from the areas highlighted in (E). The dotted line in (F) represents the principal absorption energy for low-oxidation-state (Fe^2+^ and/or Fe^0^) iron. A three-point smoothing was applied to the spectra due to low signal intensity.

Carbon *K*-edge STXM speciation maps from a hippocampal region are displayed in Figure 4A–C. Mapping at 288.3 eV, showing the overall tissue structure for this region (Figure 4A), revealed the presence of two dense structures (labeled X & Y) against a prevailing neuropil background. When mapping the same region at 287.5 eV (Figure 4B), only structure X provided high contrast, with the morphology and size of this structure being consistent with the isolated amyloid plaques we have measured previously.^28,59^ Metal speciation mapping at the calcium *L*-edge (352.6 eV)^61^ (Figure 4D) and iron *L*_3_-edge (709.5 eV) (Figure 4E) revealed the plaque to be loaded with calcium and iron of varying oxidation state, as confirmed via iron *L*_3_-edge spectromicroscopy (Figure 4F).

Iron *L*_3_-edge X-ray absorption spectra from the areas highlighted in Figure 4E are displayed in Figure 4F. Iron speciation can be determined using distinguishing spectral features at the iron *L*_2,3_-edge,^62^ as described in the Supporting Information and illustrated in the reference spectra shown in Figure S6. Area F1 was predominantly ferric (Fe^3+^) iron, whereas areas F2 and F3 were composed of mixed oxidation states of Fe^3+^ and Fe^2+^. These findings are again consistent with our previous examination of isolated amyloid plaque materials^28,59^ and strongly indicate structure X to be an amyloid plaque, thereby demonstrating the capacity and sensitivity of STXM as a tool to specifically localize and discriminate amyloid pathology against the complex multicellular surrounding brain tissue in a label-free manner. Based on the 288.3 eV absorption intensity of structure Y, this structure is likely to be a fragment of a cellular body (soma).

**Figure 5:**
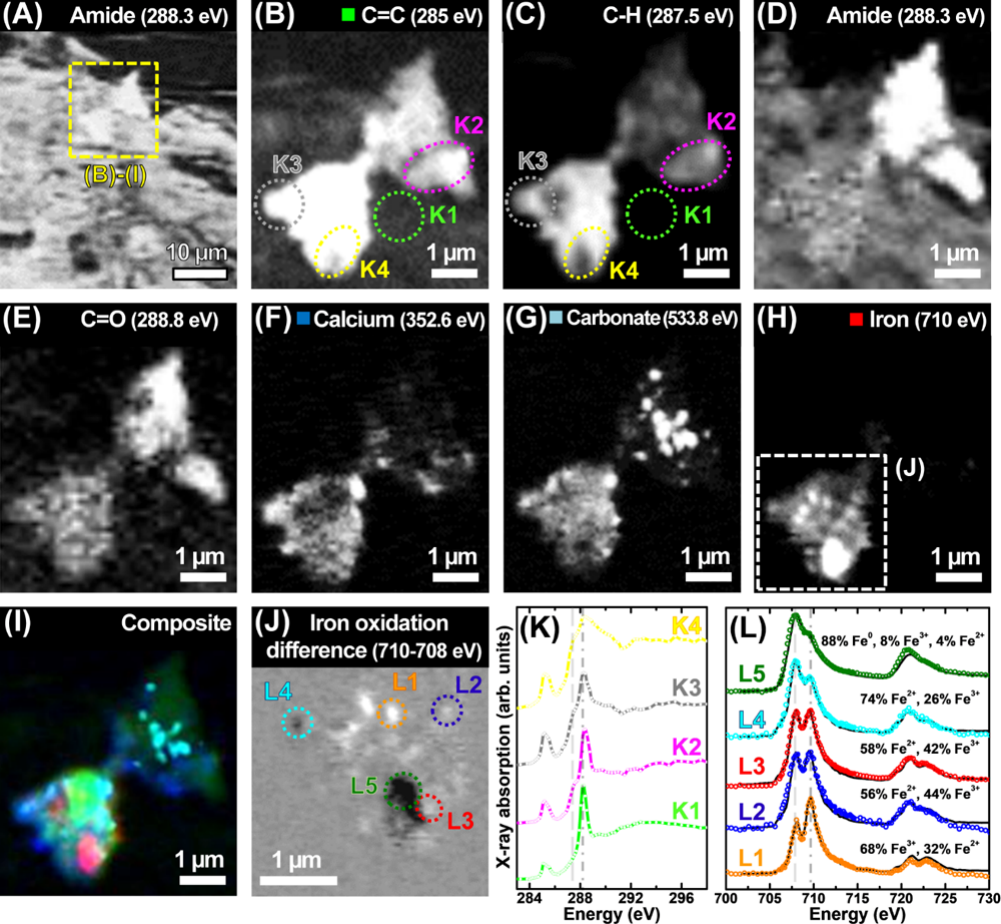
STXM examination of an amyloid plaque located within a 200 nm thick AD amygdala section. (A) Amide overview map showing tissue structure for the region containing the plaque. (B–E) Carbon *K*-edge speciation maps of the plaque. (B) C=C map, (C) C–H map, (D) amide map, and (E) C=O map. (F) Calcium *L*-edge map. (G) Oxygen *K*-edge carbonate map. (H) Iron *L*_3_-edge map. (I) Composite map showing a plaque morphology (green), calcium (blue), carbonate (sky blue), and iron (red) content. (J) High-resolution iron *L*_3_-edge oxidation state difference map of the region highlighted in (H). In the difference map, strongly absorbing oxidized iron (Fe^3+^) is shown as light contrast, and chemically reduced iron (Fe^2+^ and/or Fe^0^) is shown as dark contrast. (K) Carbon *K*-edge X-ray absorption spectra from the plaque areas highlighted in panels (B) and (C). The energies corresponding to the C–H (287.5 eV) and amide (288.3 eV) absorption features are shown by the dashed and dotted–dashed lines, respectively. (L) Iron *L*_2,3_-edge X-ray absorption spectra from the areas highlighted in (J). The energies corresponding to the reduced and oxidized states of the metals are shown by the dashed and dotted–dashed lines, respectively.

**Figure 6:**
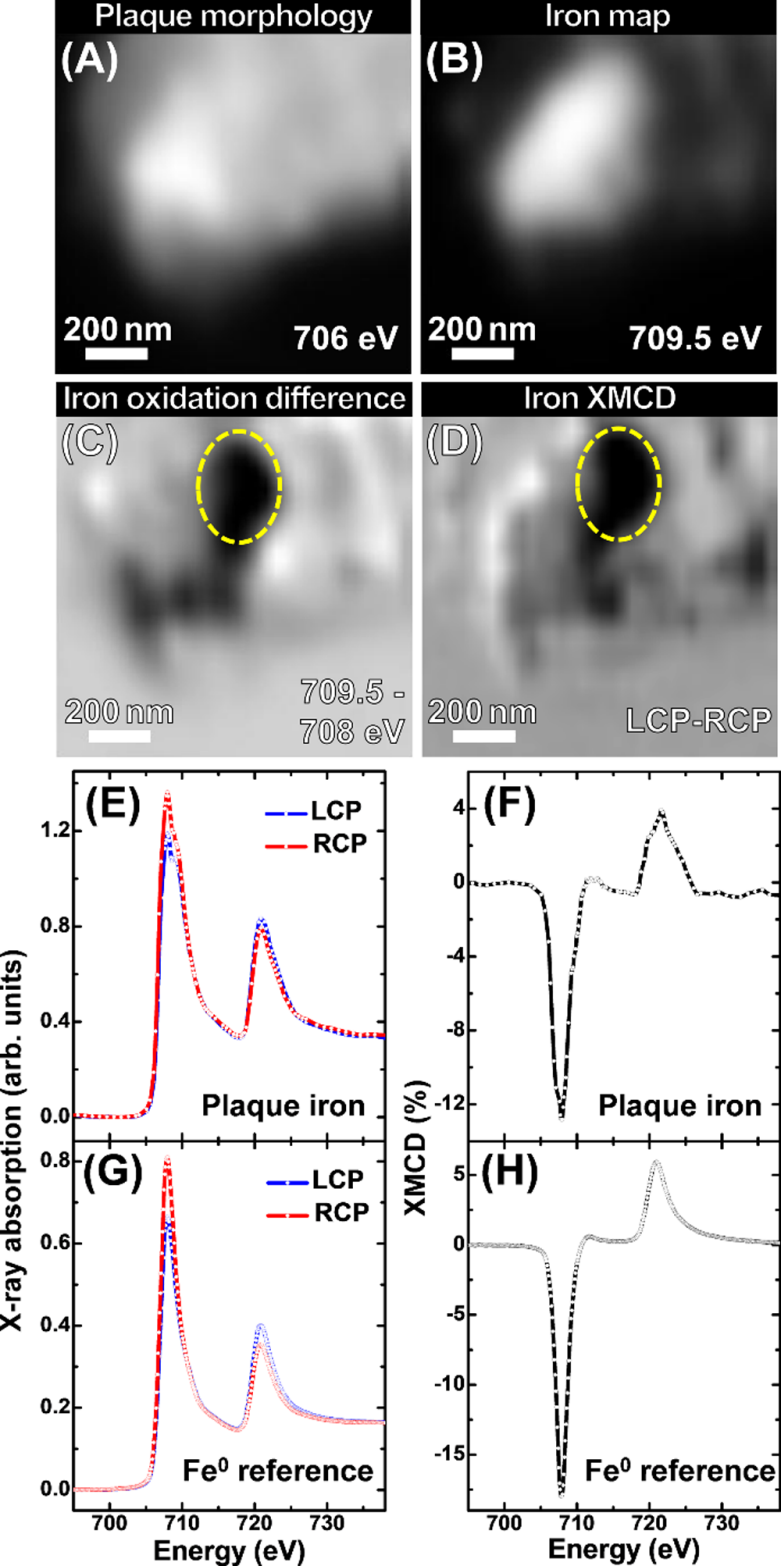
High-resolution STXM XMCD measurements of the bottom-most amyloid plaque iron region shown in Figure 5, incorporating area L5. (A) Single energy STXM image showing the plaque morphology. (B) Iron *L*_3_-edge map. (C) Iron *L*_3_-edge oxidation state difference map. (D) Iron *L*_3_-edge XMCD map, where areas of bright and dark contrast represent the presence of magnetic iron. (E) LCP and RCP iron *L*_2,3_-edge X-ray absorption spectra and (F) XMCD spectra from amyloid plaque iron area L5, also highlighted in yellow in maps (C) and (D). (G) LCP and RCP iron X-ray absorption spectra and (H) XMCD spectra from a thin Fe^0^ reference film.^64^

**Figure 7:**
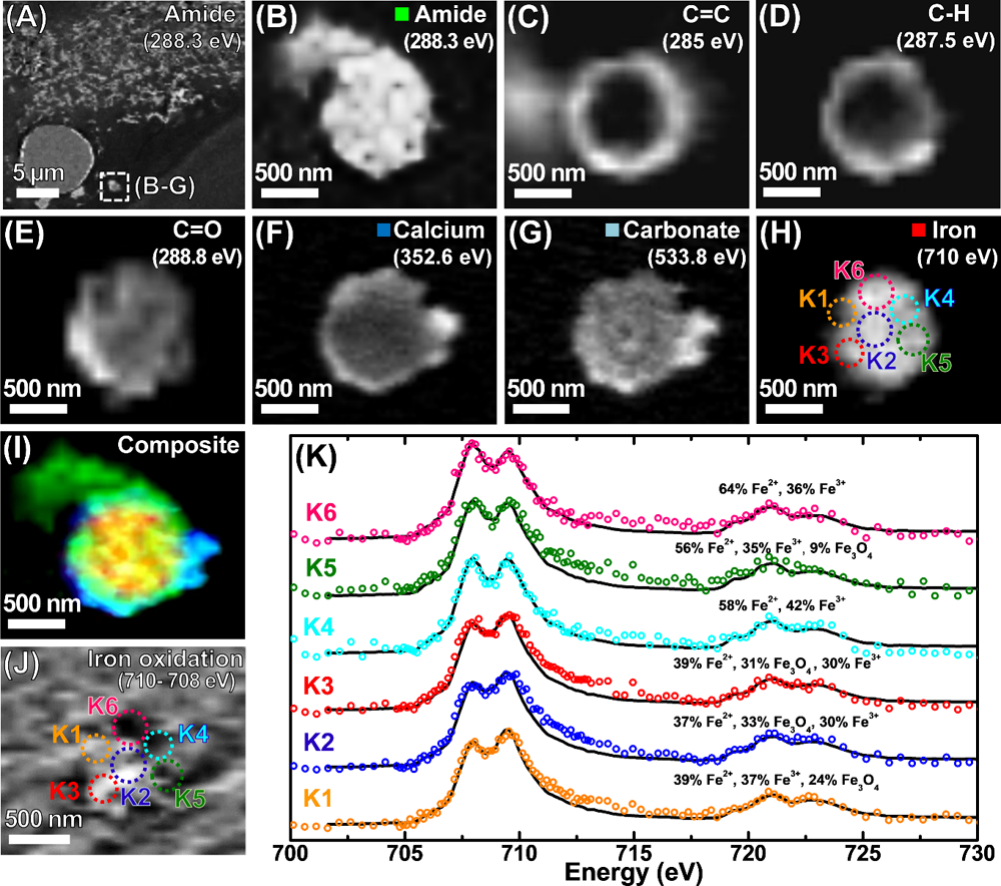
STXM examination of an amyloid plaque located within a 200 nm thick amygdala section from a neurologically healthy control. (A) Amide overview map showing tissue structure for the region containing the plaque. (B–E) Carbon *K*-edge speciation maps of the plaque. (B) Amide map, (C) C=C map, (D) C–H map, and (E) C=O map. (F) Calcium *L*-edge map. (G) Oxygen *K*-edge carbonate map. (H) Iron *L*_3_-edge map. (I) Composite map showing a plaque morphology (green), calcium (blue), carbonate (sky blue), and iron (red) content. (J) Iron *L*_3_-edge oxidation state difference map. (K) Iron *L*_2,3_-edge X-ray absorption spectra from the areas highlighted in (H and J).

This label-free imaging methodology was utilized to locate further plaques within the tissue sections of AD amygdala (Figures 5, 6, S7–S16) and hippocampus (Figures S17 and S18) and neurologically healthy aged amygdala (Figures 7 and S19). The label-free imaging methodology was further validated via correlative Congo red staining of tissue regions examined under STXM where structures that absorbed strongly at 287.5 eV were shown to display “apple-green” birefringence (indicating the presence of amyloid material) when stained with Congo red (Figure S18). As with the isolated plaque cores, the 287.5 eV absorption feature in the plaques was not an artifact of X-ray beam saturation nor sample density, as demonstrated in Figure S20.

Figure 5 shows an example of an amyloid plaque within a 200 nm thick section of the AD amygdala. As with the hippocampal region shown in Figure 4, the amyloid plaque absorbed strongly at 287.5 eV, whereas the surrounding neuropil did not (Figure 5C), allowing the plaque to be clearly discriminated against the tissue background.

Examination of this amygdala region across the entire carbon *K*-edge (Figure 5K) showed that the neuropil had a carbon X-ray absorption spectrum (from selected area K1) entirely consistent with the tissue reference spectrum shown in Figure 2E. Likewise, the carbon spectra of the amyloid plaque (areas K2–K4 in Figure 5B, C) were consistent with the isolated plaque core shown in Figure 3, with a broad C 1s → σ*/Rydberg C–H transition at 287.5 eV observed in all areas and higher energy 288.7 eV C 1s → π* C=O transition evident in areas K2 and K4. Speciation mapping at the calcium *L*-edge (Figure 5F), oxygen *K*-edge (Figure 5G), and iron *L*_3_-edge (Figure 5H) showed the amygdala plaque to contain deposits of calcium, carbonates, and iron, consistent with our previous findings from isolated amyloid plaques.^28,59^ Some plaque regions showed calcium and carbonate colocalization, suggesting the presence of calcium carbonate. However, the distribution of both calcium and carbonates spread beyond these areas of colocalization, suggesting that multiple calcium and carbonate species are present.

High-resolution iron oxidation difference mapping (Figure 5J, from the selected area in 5H) showed a dramatic variation in the oxidation state of the plaque iron, with evidence of oxidized (light contrast) and low-oxidation-state (dark contrast) iron deposits. X-ray spectromicroscopy performed across the entire *L*_2,3_-edge (Figure 5L) confirmed this variation in the plaque iron oxidation state within a single amyloid plaque. By analyzing these spectra and specific iron references, we determined that area L1 was primarily ferric (Fe^3+^), areas L2 and L3 represented mixed Fe^3+^/Fe^2+^ phases; area L4 was predominantly ferrous (Fe^2+^) and area L5 was consistent with predominantly elemental metallic (i.e., zero-oxidation-state; Fe^0^) iron, with a small oxidized component. See Figure S6 (pink trace) for reference Fe^0^ iron *L*_2,3_-edge X-ray absorption spectrum.

As described previously,^28^ elemental iron is one of only three transition metals that display strong ferromagnetism. Therefore, to confirm the presence of elemental metallic Fe^0^ in the plaque, the fundamental magnetic properties of area L5 from Figure 5 were probed by magnetically sensitive XMCD measurements (Figure 6).

Iron *L*_3_-edge XMCD mapping of the area (Figure 6D) showed strong dark contrast, indicating that this iron inclusion is strongly magnetic. The corresponding circular-polarization-dependent X-ray absorption and XMCD spectra for this plaque iron, along with those from a pure elemental Fe^0^ film standard, are shown in Figure 6E–H. Circular polarization-dependent X-ray absorption (Figure 6E) and XMCD spectra (Figure 6F) obtained from area L5 showed strong dichroism, resulting in a negative (∼13%) XMCD peak at 708 eV and a smaller positive peak (<5%) at 721 eV. The shape and intensity of this magnetic dichroism strongly resemble the spectra of the ferromagnetic metallic Fe^0^ film standard (Figure 6G,H), albeit with small differences due to the ca. 12% oxidized component of the plaque iron. It should be noted that no evidence of the complex three-peak XMCD spectra typical for the ferrimagnetic biominerals magnetite and maghemite was observed,^63^ suggesting that the oxidized component of this amyloid plaque iron is nonmagnetic.

STXM analyses of 11 further amyloid plaques from tissue sections of AD amygdala and hippocampus are shown in Supporting Information Figures S7–S18. All plaques examined over the carbon *K-*edge, provided carbon X-ray absorption spectra with a broad C–H absorption feature at 287.5 eV. Multiple plaques contained regions with a positively shifted C=O peak position when compared to the tissue parenchyma. All plaques were found to contain calcium and carbonates consistent with our previous examination of isolated amyloid plaque cores.^34^ Eight of the 10 plaques (80%) examined at the iron *L*-edge contained detectable iron deposits, with seven of these plaques providing evidence of chemically reduced iron deposits. Two amyloid plaques were found to contain deposits of potassium detectable by both STXM speciation mapping and spectroscopy (Figures S8 and S14).

### STXM Analysis of Amyloid Plaques within Disease-Free Control Tissues

Despite being recognized as a hallmark pathological lesion of AD, amyloid plaques are also known to develop in the brains of neurologically healthy individuals as a function of normal brain aging,^65^ albeit in lower abundance relative to the disease state. We therefore applied STXM to locate and perform biochemical analysis on amyloid plaques within the amygdala tissue from an aged neurologically healthy control subject.

In total, two suspected amyloid plaques (shown in Figures 7 and S19) were located within the tissue classified post-mortem from a neurologically healthy individual when examined by STXM. Carbon *K*-edge STXM speciation mapping of the plaque in Figure 7 showed the plaque to be carbon dense, containing regions of C=C (Figure 7C) and C–H accumulation (Figure 7D) as was the case with amyloid plaques located within the brain tissue from confirmed AD cases. In this instance, C–H and C=C groups were found to be preferentially accumulated in the outer boundaries of the plaque while also infiltrating the core at lower levels. A similar distribution pattern has previously been observed for lipids in plaques from humans and transgenic AD mouse models.^56,66^

Consistent with the amyloid plaques from AD tissues shown in Figures 4, 5, and S7–S17, speciation mapping at the calcium *L*-edge (Figure 7F), oxygen *K*-edge (Figure 7G), and iron *L*_3_-edge (Figure 7H) showed this plaque to be loaded with calcium, carbonates, and iron. Iron oxidation difference mapping (Figure 7J) and *L*_2,3_-edge spectromicroscopy (Figure 7K) showed nanoscale variation in the amyloid plaque iron oxidation state, including the presence of low-oxidation-state iron regions, in keeping with the observation of chemically reduced iron in amyloid plaques from AD tissues.

The additional plaque from the control tissue shown in Figure S19 also contained calcium, carbonates, and iron. Spectromicroscopy and fitting performed over the iron *L*_2,3_-edge showed the plaque iron oxidation state to be predominantly ferric, with minor ferrous contributions.

The presented results establish the use of synchrotron-based X-ray spectromicroscopy to visualize nanoscale variations in the chemical composition of the human brain tissue using a new methodology for high-resolution label-free chemical analysis of amyloid pathologies within brain tissue sections made possible through the chemical and magnetic specificities of STXM. This innovative X-ray spectromicroscopy approach requires no staining of the sample with either dyes or heavy metal contrast agents (e.g., osmium tetroxide and uranyl acetate) used in conventional imaging techniques, thereby providing an unprecedented insight into the biochemistry of amyloid plaques and the AD brain.

Visualization of amyloid plaques within brain tissue sections was achieved by exploiting a characteristic feature in plaque-derived carbon *K*-edge X-ray absorption spectra. This feature can be attributed to the presence of aliphatic hydrocarbon chains,^47,52,53^ consistent with the accumulation of lipids around/and within the plaques. Further chemical analysis showed that amyloid plaques contained calcium, carbonates, and iron in varying oxidation states. Remarkably, iron chemistry was found to vary over submicron length scales, with oxidized (i.e., Fe^3+^) and low-oxidation-state iron, including ferromagnetic elemental iron (Fe^0^) being observed. These findings strongly implicate amyloid-β in the formation of elevated levels of potentially redox-active, low-oxidation-state iron phases in the human brain. The ability to spatially separate different metal phases was made possible through the combined chemical sensitivity and nanoscale imaging spatial resolution of STXM, highlighting the importance of chemical nanoimaging when examining metal biochemistry in living systems.

Carbon *K*-edge spectra from amyloid plaques contained a broad absorption feature, absent in carbon *K*-edge spectra from the surrounding tissue, which can be attributed to C–H 1s → σ* transitions present in high quantities in lipid hydrocarbon chains.^47,52,53^ This feature was first identified through the examination of the isolated amyloid plaque material and was subsequently used to distinguish amyloid plaques from the surrounding tissue matrix within brain tissue sections. The prominence of this feature was somewhat surprising as carbon *K*-edge σ* transitions are more broad and lower in intensity when compared to their π* transitions (e.g., the white line amide peak at 288.3 eV) which typically dominate spectra collected at this absorption edge. The large peak intensity of the amyloid plaque 287.5 eV 1s → σ* feature therefore suggests a localized high concentration of C–H bonds, such as those found in the aliphatic chains of hydrocarbons. Importantly, this absorption feature was confirmed not to be an artifact of X-ray beam saturation due to sample density (Figures S2 and S20).

Carbon spectra from numerous plaque regions also contained a 288.7 eV C=O 1s → π* peak feature indicative of simple carbonyl groups, as opposed to the anticipated red-shifted 288.3 eV C=O 1s → π* amide peak that would normally be expected from protein-rich structures.^46^ The presence of carboxyl and aliphatic hydrocarbon absorption features indicates the presence of lipids. Both of these absorption features are prominent in reference carbon *K*-edge phospholipid spectra,^54,67^ with select carbon spectra collected from isolated amyloid plaque cores bearing clear similarities to phospholipid references.

The observation of lipids in amyloid plaques from both human AD tissues and transgenic mouse models of the disease has previously been reported,^55,56,58^ with lipid accumulation being shown to occur in the dense cored plaque subtype and not the diffuse subtype. This is consistent with our observation of enhanced C–H and C=O carbonyl absorption features from isolated amyloid plaque cores. Amyloid structures visualized within brain tissue sections using 287.5 eV absorption intensity shown here may therefore predominantly represent dense cored plaques (rather than diffuse or coarse-grained plaques). Lipid absorption features provide an explanation for the spectral features we reported previously when examining the carbon chemistry of amyloid plaques within the cortical tissue from the transgenic APP/PS1 mouse line.^64^ This spectrum displayed enhanced X-ray absorption in the 287.5 eV region, suggesting that plaques in this mouse model may also accumulate lipids.

Potential sources of lipids accumulated within amyloid plaques have been discussed in depth, with multiple plausible mechanisms for the coalescence of lipids and amyloid being suggested. Examples of lipid sources include the following: (i) phospholipid membranes, including vesicle membranes and dystrophic neurites. In the former case, amyloid-β fibrils have been shown to extract lipids actively in a detergent-like mechanism during its aggregation. (ii) Microvesicles from microglia, which have been shown to adhere to amyloid plaques. Microglia are known to be activated in AD and then surround amyloid plaques and have a role in sequestering amyloid through phagocytosis. (iii) The lipid transporter Apolipoprotein E (ApoE) and its lipid cargo are known to colocalize with amyloid plaques.^55,56,58^

In relation to disease pathogenesis, lipids and lipoproteins can influence amyloid aggregation and also influence the extent to which amyloid-β oligomers could be released from fibrillar plaques. These soluble oligomers have been shown to convey toxic effects that may contribute to the toxic mechanisms of amyloid plaques in the AD brain. Amyloid-β can also insert into the cell membrane forming pore-like structures with the potential to disrupt normal cellular functions.^68,69^ However, it was not our intention here to investigate lipids and associated lipoproteins as a contributing factor in the evolution of AD, and this subject has been discussed in detail elsewhere.^70^

Plaques identified within AD tissues from different affected areas and neurologically healthy tissues using this label-free approach frequently exhibited extensive carbonate and calcium deposits, which are consistent in their properties with isolated amyloid plaque cores that we have described previously.^34^ The levels of both calcium and carbonates greatly exceeded the surrounding brain parenchyma, indicating that a process of active calcium and carbonate deposition or biomineralization may be occurring during the formation of amyloid plaques. From the calcium *L*-edge and oxygen *K*-edge STXM mapping performed in this study, it was not possible to determine the origin of the observed calcium. However, calmodulin and other calcium binding proteins (e.g., lithostathine) or pools of mitochondrial and extracellular Ca^2+^ may represent calcium sources.^59,71,72^ As differing calcium minerals provide distinct X-ray absorption spectra,^73^ more detailed X-ray spectromicroscopy measurements performed over the entire Ca *L*_2,3_-edge may provide further information on the source of amyloid plaque calcium. Exploiting crystal field splitting and X-ray linear dichroism may be useful in this regard by providing information on the crystallinity and crystal orientation of the calcium phases.^74,75^

Calcium is integral to a multitude of signaling pathways in the brain.^71,72,76^ The accumulation and binding of calcium within amyloid plaques^77^ may have a detrimental effect on these pathways by reducing the bioavailability of the metal. Previous studies have shown amyloid-β to negatively impact calcium-dependent signaling pathways impairing neuronal function.^76^

Overall, iron *L*_3_-edge STXM speciation mapping performed on 13 plaques showed that 11 contained detectable deposits of iron. Examination of the iron chemical state across the *L*_2,3_-edge revealed nanoscale variations in the plaque iron oxidation state in both AD and control tissues, including chemical variation within the same plaques. Plaque iron oxidation states ranged from entirely ferric phases (Fe^3+^) to mixed-valence, ferrous (Fe^2+^), and elemental metallic ferromagnetic Fe^0^, as confirmed via magnetically sensitive XMCD measurements. The presence of low-oxidation-state iron phases including elemental ferromagnetic iron within amyloid plaques in AD tissue sections is consistent with our examination of isolated amyloid plaque cores.^28,59^ The observation of elemental ferromagnetic iron is further noteworthy as without a suitable coating we would expect this iron to oxidize rapidly when exposed to an aerobic environment. While it must be acknowledged that thin (∼3 nm) self-passivating iron oxide layers can prevent the total oxidation of Fe^0^ nanoparticles,^78^ our own STXM measurements of air-exposed Fe^0^ nanoparticle standards embedded using the same resin as the tissue samples showed these standards to become oxidized during sample preparation and examination, such that XAS and XMCD spectra from these particles resembled the mixed Fe^2+/3+^ iron phase magnetite and its ferric oxidation product maghemite (see Figure S7; Everett et al.^28^). These observations suggest that low-oxidation-state metals located within amyloid plaques are protected from oxidation.

As with calcium, the origin of the iron accumulated within the amyloid plaques could not be determined here using STXM. We previously discussed multiple endogenous iron sources that may be relevant to amyloid-β/iron interaction in the brain.^59^ Examples include ferritin, transferrin, labile iron pools, hemosiderin, and disrupted mitochondria.^59,79^ Multiple plausible mechanisms for the formation of elemental metallic metal phases in the human brain were discussed in our previous publication.^28^

A further possibility is that low-oxidation-state iron particulates, including elemental Fe^0^, could be sourced from exogenous sources such as airborne metal-rich ultrafine particulate matter (<200 nm diameter).^80^ The potential for nanoparticulate air pollution to contribute to neurodegenerative mechanisms is an area identified by the 2018 report of the Lancet Commission on Pollution and Health as being a top research priority.^81^ Ultrafine particles may enter the brain via a variety of pathways such as the olfactory mucosa (bypassing the blood–brain barrier), lung, and gut-brainstem axis and can contain large concentrations of combustion-derived components, such as organic carbon compounds and small metal particles.^82,83^ However, we would expect these metal particles to become oxidized prior to reaching the brain. Thus, even if the low-oxidation-state iron nanodeposits observed within amyloid plaques originated directly from an exogenous source, these particles would have to undergo additional chemical reduction during their incorporation into the plaque, in which their (chemically reduced) oxidation state was then stabilized.

The accumulation of low-oxidation-state iron in amyloid plaques may contribute to increased brain redox burdens and associated brain cell loss in AD.^23,24,84−90^ The presence of these phases may also explain the apparent ability of amyloid-β to induce ROS-mediated neurotoxicity^91−93^ and suggests that redox burdens may be dictated by local iron chemistry rather than absolute iron concentration.^94^ The sequestering of iron by amyloid plaques may further induce neurotoxicity indirectly by removing and delocalizing pools of bioavailable iron needed for physiological brain processes.^95^

Paradoxically, the incorporation of low-oxidation-state, redox, and catalytically active iron within aggregated amyloid structures may convey an antioxidant mechanism by preventing metal-catalyzed oxidation damage to local tissue structures. In this scenario, any subsequent dissolution of plaques may create local sources of toxic reactive iron species in the brain. This scenario is consistent with the hypothesis that amyloid plaque formation may be a physiological response to toxic species rather than a driver of pathological *damage.*(93,96)

Precise chemical speciation of metal phases associated with the development of AD may prove crucial in the development of viable metal-targeting technologies intended for disease diagnosis, monitoring, and treatment. Amyloid plaques rich in iron and calcium can be visualized using MRI,^97^ suggesting that the incorporation of iron and the biomineralization of amyloid plaques could have utility as endogenous biomarkers for disease diagnosis and staging. The ferromagnetic properties of elemental Fe^0^ are distinct from the magnetic properties of physiological iron oxide forms, which could be further exploited to image this type of iron selectively. Low-oxidation-state iron, including Fe^0^ which presently has no known physiological function and that we have revealed is associated with amyloid deposition in human tissue, may represent an innovative target for AD therapies intended to lower redox burdens by alleviating iron-associated toxicity.^36^ It is critical that these iron-targeting strategies do not perturb essential iron-dependent physiological processes. Understanding the fundamental role of amyloid plaque formation in human tissue is essential if strategies such as targeting amyloid-β-iron interactions in the brain are to be explored. The details of these processes in the living brain will determine whether there is scope to modulate the potential neurotoxicity arising from this interaction, thereby affecting disease progression.

The label-free identification of amyloid plaques, as developed in this study, opens up a variety of follow-up investigations to understand further the biochemistry of human brain tissues and AD pathologies. Crucially, by avoiding the use of conventional chemical stains to identify amyloid plaques, their associated chemistry can be probed without having already been disrupted by staining.

It is now necessary to extend the range of elemental absorption edges examined using X-ray spectromicroscopy to include other metals and metalloids implicated in neurodegenerative disease,^13,98−100^ along with low-atomic number metal-binding elements such as those incorporated in metalloproteins. It is, however, important to recognize that element profiling alone via X-ray fluorescence (XRF) or other methods is not sufficient to fully understand the chemical composition of these sample types owing to the variety of different chemical compounds observed for many given elements. As the chemical oxidation state of metal elements dictates their reactivity, this information (beyond concentration alone) is required to more fully establish the role of metals in human disease. Complementing nanofocus XRF mapping with X-ray spectromicroscopy analysis will therefore be useful in this regard by providing an elemental profiling of AD tissues and pathologies to inform the selection of suitable target elemental absorption edges for additional speciation analysis.

Another consideration for subsequent studies will be the chemical profiling of amyloid plaques of differing subtypes. The 287.5 eV absorption feature used to map plaques in this work was identified through the examination of isolated amyloid plaque cores derived from dense, mature plaques. It has not yet been established whether this feature is also present in other plaque types such as diffuse, primitive, and coarse-grained plaques. This is an important consideration when interpreting the data sets presented here as differing plaque types induce unique cellular responses including microglial and astrocyte activation and neuroinflammation.^101^ Furthermore, our previous examination of both in vitro amyloid structures formed in the presence of ferric iron and amyloid plaques from a transgenic APP/PS1 mouse model of AD indicate the iron oxidation state (including the presence of chemically reduced and metallic elemental phases) to be linked to amyloid plaque type.^32,49,102^ A similar pattern may occur in human AD tissues and warrants further investigation. Evidence suggests that differing plaque subtypes accumulate preferentially in the AD brain dependent on the ApoE4 status and whether the disease is early or late onset.^101^ Thus, understanding how metal biochemistry differs across plaque subtypes may prove vital in understanding how altered metal homeostasis contributes to disease pathogenesis.^101^

Presently, synchrotron X-ray microscopy experiments in this field are constrained by limited access to beamtime (via competitive proposal access to large facilities) and the time-consuming nature of the measurements. Efficient visualization of amyloid plaques, enabled by the label-free screening processes demonstrated here, will optimize use of future synchrotron experiment time and maximize the number of samples that can be analyzed in each allocated experimental facility shift. Forthcoming upgrades of globally used synchrotron light sources^103^ will facilitate increasingly rapid measurement in a finite allocation of time, providing scope to further strengthen experiment design with higher sample throughput.

The STXM methodology presented here can be readily adapted to examine a range of biological materials and is not limited to the examination of brain tissues. Excitingly, ongoing development of cryogenic measurement capabilities at many synchrotron beamlines will create opportunities for the examination of vitrified biological specimens.^104^ A cryoSTXM approach removes the requirement for the resin embedding of tissue sections, offering unprecedented insight into the native-state biochemistry and ultrastructure. Performing STXM under cryogenic conditions also protects against X-ray damage induced by sample measurement at high X-ray doses,^105^ allowing for increased tolerances to X-ray radiation which will improve data quality.

In conclusion, we have presented novel X-ray spectromicroscopy methodology for in situ label-free chemical analysis of amyloid pathologies within the human brain tissue, made possible through the nanoscale chemical specificities of STXM. This approach has enabled precise imaging and chemical speciation of organic and inorganic amyloid plaque components, without the need for contrast agents, dyes, or aldehyde fixatives used in conventional imaging techniques. Plaques were shown to contain elevated levels of calcium and iron compared to the surrounding tissue parenchyma, with evidence that the plaques incorporate low-oxidation-state iron, including elemental ferromagnetic nanodeposits. The reactivity of metallic iron differs from its oxide forms and may have a significant bearing on metal toxicity and oxidative burden in the AD brain. These insights challenge our understanding of disease etiology with a potential to improve therapeutic strategies for AD.

## Methods

Human brain tissue/tissue-derived material was obtained from four AD cases and a further neurologically healthy aged control subject. The brain bank tissue was provided to the study and used under UK ethical approval 07/MRE08/12 and USA IRB 03–00–26.

### Embedding of the Human Brain Tissue

Human brain tissue was embedded and sectioned for STXM as previously described in Brooks et al.^50^ Frozen brain tissue that had not been chemically fixed was acquired from Newcastle Brain Tissue Resource (Newcastle, UK) and the Canadian Brain Tissue Bank (Canada). Amygdala and putamen tissue samples from a confirmed AD case and hippocampal tissue from a second confirmed AD case (both Braak Stage VI) were examined along with the amygdala tissue from an age-matched neurologically healthy control case.

Frozen tissue samples were cut into cubes (<1 cm^3^) using a ceramic blade to prevent metal contamination, within a cryotome operating at −16 °C. Tissue cubes were dehydrated through an ethanol series (40–100% dry) in a Class II laminar flow hood at room temperature. Dehydrated tissues underwent a six-stage resin embedding procedure using the STXM-compatible resin composed of an equimolar mixture of trimethylolpropane triglycidyl ether: 4,4′-methylenebis(2-methylcyclohexylamine). This resin, successfully used by our group as reported in our previous examination of biological tissues using synchrotron X-ray spectromicroscopy,^28,34,49,50,59,106,107^ contains no carbonyl or aromatic groups, making it an ideal embedding substrate when examining protein-rich structures due to the lack of strong π* spectral features at the carbon *K*-absorption edge that overlap with principal absorption features arising from proteins.^46^ Initially, tissue samples were immersed in 75% dry ethanol and 25% resin. The proportion of resin was increased in increments of 25% every two h with continuous mixing, with two further hourly changes of 100% resin for infiltration and removal of any residual ethanol. Resin polymerization was performed at 60 °C over 24 h. The introduction of formalin or aldehyde fixatives was avoided in all sample types to prevent metal leaching and alteration of the metal mineral composition in the tissue sample materials. The absence of aldehyde fixation via protein cross-linking and the use of ethanol dehydration in the embedding process may have caused some loss in fine tissue ultrastructure and tissue shrinkage, reducing the apparent size of biological features, including plaques and cells, when compared to their hydrated state. Semithin sections (200 or 500 nm thickness) of the embedded brain tissue were cut using a Reichert-Jung Ultracut microtome operating with a nonmetallic diamond or sapphire blade to minimize the risk of metal contamination during the sectioning process. For STXM experiments, tissue sections were deposited onto TEM grids (Agar Scientific; 100 mesh), and these grids were mounted onto STXM sample plates. In select instances, consecutive sections were obtained to enable correlative Congo red staining and STXM analysis of the same tissue region. 500 nm thick sections for Congo red staining were deposited onto TEM grids.

### Isolation and Embedding of Amyloid Plaques

Amyloid plaque cores were prepared for STXM analysis as previously described in Everett et al.^28,59^

The brains of two confirmed Braak stage VI AD cases were removed at autopsy 5 h post mortem, divided into half, and cut into 1 cm slices before being stored in an ultrafreezer at −70 °C. Slices from the frontal and temporal lobes were thawed, and the gray matter was isolated through the removal of the white matter, blood vessels, and meninges. Isolated gray matter was homogenized by heating to 95 °C in the presence of 2% SDS in 50 mM Tris-buffer. Remaining large tissue debris was removed through filtration (100 μm pore size), and the homogenate was pelleted through centrifugation at 800 rpm for 15–30 min. Further homogenization was performed though the addition of 0.1% (w/v) SDS, 150 mM NaCl, and 0.02% NaN_3_ (w/v) before filtration (35 μm pore size) and pelleting through centrifugation at 1000 rpm for ca. 30 min. Amyloid plaque cores were isolated from the filtered tissue homogenate via ultracentrifugation at 20,000 rpm in a sucrose gradient (1.8–1.2 M sucrose in a 0.1% (w/v) SDS, 150 mM NaCl, and 0.02% (w/v) NaN_3_ solution). The resulting fractions were recovered with 0.1% (w/v) SDS, 150 mM NaCl, and 0.02% (w/v) NaN_3_ and finally concentrated via centrifugation at 1200 rpm.

A 40 μL aliquot of the concentrated amyloid plaque core material in suspension was transferred into a centrifugal concentrator (Corning Spin-X UF; 40 kDa cutoff) and spun at 6690 rpm for 10 min before being dehydrated through an ethanol series (100 μL; 40–100% dry), with waste ethanol being removed at each step through centrifugation at 6690 rpm for 10 min. Following ethanol dehydration, the amyloid plaque core material was embedded in the resin according to the protocol described above. Resin polymerization was performed at 60 °C over 24 h. Semithin sections of embedded amyloid plaques were cut to either 500 or 200 nm thickness, with a Reichert-Jung Ultracut microtome operating with a diamond blade. 200 nm-thick sections containing embedded amyloid plaque cores were deposited onto TEM grids (Agar Scientific; 100 mesh), and these grids were in turn mounted onto sample plates for STXM examination. 500 nm thick sections were deposited onto TEM grids and set aside for Congo red staining.

### Congo Red Staining

500 nm thick sections of isolated amyloid plaque cores or AD tissue were stained for amyloid structures using a 1% Congo red solution. Sections were stained for ca. 30 min at room temperature, and excess stain was removed with deionized H_2_O. Stained sections were examined for birefringence under cross-polarized light using an Olympus IX51 microscope (60× objective lens).

### Scanning Transmission X-ray Microscopy

X-ray spectromicroscopy experiments were performed using STXM at the Advanced Light Source (ALS; Lawrence Berkeley National Laboratory, CA, USA) beamline 11.0.2, Diamond Light Source (DLS; Oxfordshire, UK) beamline I08 and MAX IV (Lund, Sweden) SoftiMAX beamline. For STXM schematic, see Figure S21 of the Supporting Information. Focused X-ray spot sizes were ≤50 nm at all sources. As described in our previous work,^28^ X-ray photon doses were kept as low as reasonably possible while ensuring a sufficient signal:noise ratio to minimize photon-dose effects and thereby negate disturbances to native sample chemistry. This was achieved by carefully controlling sample exposure time (dwell time per image pixel) as well as reducing X-ray intensity by using narrow beamline exit slits. For further information on this procedure, see the Supporting Information of Everett et al.^28^ Where multiple images of an ROI were taken at different energies, images were aligned to a common feature using cross-correlation analysis, compensating for any movement in the sample or X-ray beam position that may have occurred during scanning.

#### Speciation Mapping

To map the distribution of chemical species within the sample materials, paired images were acquired: one at the energy corresponding to the spectral feature of interest and a second at an off-peak energy a few eV from the feature. Raw X-ray absorption intensities were normalized to the incident X-ray beam by conversion to optical density using appropriate background regions. Following conversion to optical density, the off-peak image was then subtracted from the peak image to create a difference map, providing a chemical speciation image over the region of interest for the chosen spectral feature. This method of speciation mapping removes artifacts from the embedding resin, revealing a true representation of the chemical distribution arising from the sample material.

By finely tuning the energy of the incident X-ray beam, distribution maps showing distinct chemical states were generated by exploiting the preferential absorption of X-rays of differing energies by specific compounds. The carbon and oxygen X-ray absorption *K-*edges were used to visualize the organic components of the sample material. Metal maps were created at the calcium *L*-edge and iron *L*_3_-edge showing calcium and iron distribution, respectively.

Examples of the speciation mapping process are shown in Supporting Information Figures S22 and S23, where the oxygen *K*-edge energy corresponding to the 1s → π* transition for COO/COOH/CONH_2_ groups (ca. 532 eV) is used to visualize the tissue structure in a 500 nm thick tissue section (Figure S22),^45^ and the ferric iron *L*_3_-edge absorption feature (710 eV) is used to show iron distribution within a suspected amyloid plaque in a 200 nm thick section (Figure S23).^62^ Additional iron oxidation state difference maps were created by subtracting images taken at energies corresponding to a chemically reduced Fe^2+^/Fe^0^ state (708 eV) from the oxidized Fe^3+^ state (709.5 eV).^62^ These maps provide a qualitative distribution of different iron oxidation states, with reduced phases showing as dark contrast and oxidized phases showing as light contrast (see also Supporting Information Figure S23F).

#### X-ray Absorption Spectra

X-ray absorption spectra providing detailed information regarding the chemical state of the sample material were obtained from a series of images (called a “stack”) acquired over a desired element absorption edge. Using this approach, X-ray absorption spectra can be generated from a selected single pixel or group of pixels within an image, allowing the chemical composition of highly localized (<50 nm in this instance) regions of interest to be determined (see Supporting Information Figure S24).

Stacks were acquired over the carbon *K*-edge (280–320 eV) and iron *L*_2,3_-edge (700–740 eV). For stack measurements, the dark count (background noise attributable to the beamline) was subtracted prior to generation of the X-ray absorption spectra.

#### STXM-XMCD

To establish the magnetic state of selected iron inclusions detected within the sample materials, magnetically sensitive X-ray magnetic circular dichroism (XMCD) measurements were performed.

XMCD measurements were performed by inserting a permanent magnet (a NdFeB ring magnet array, allowing X-ray transmission) into the rear face of the STXM sample holder and mounting the sample to the front face of the holder (see Figure 4 of Everett et al.^28^). This created a magnetic field strength of ∼250 mT perpendicular to the sample surface, which we have previously shown to induce a sufficient degree of magnetic polarization in both iron mineral standards and magnetic iron inclusions within amyloid plaque pathologies to measure magnetic dichroism.^28,34^

To generate XMCD spectra from magnetized samples, X-ray absorption spectra were recorded from paired stacks performed over the iron *L*_2,3_-edge using both left and right circularly polarized light. The dichroism is revealed as the difference spectrum obtained by subtracting the X-ray absorption spectra obtained using right circularly polarized (RCP) light from the equivalent spectra obtained using left circularly polarized (LCP) light (see Supporting Information Figure S25).

To visualize magnetic iron deposits showing strong magnetic polarization, we also created XMCD maps. These maps were obtained by averaging five images collected over the Fe^2+^/Fe^0^ peak energy position (∼708 eV) for both RCP and LCP measurements. The averaged RCP image was then subtracted from the equivalent averaged LCP image to yield an XMCD difference map, with areas of bright or dark contrast indicating significant dichroism effects (Supporting Information Figure S26).

#### STXM Data Processing

STXM data were processed using the aXis 2000 software package (http://unicorn.mcmaster.ca/aXis2000.html). Grayscale X-ray microscopy images were converted to false color and recombined as overlays to create pseudocolored composite images using ImageJ.

#### Fitting of X-ray Absorption Spectra

Where there was sufficient absorption intensity, the relative proportions of different iron phases contributing to experimental iron *L*_2,3_-edge X-ray absorption spectra were estimated by fitting the measured spectra to standards of Fe^3+^, Fe^2+^, Fe_3_O_4_ (magnetite), and Fe^0^, using a nonlinear least-squares fitting procedure as first described in Everett et al.^34^

The four reference iron *L*_2,3_-edge X-ray absorption spectra obtained from ferritin, FeCl_2_, magnetite, and a thin Fe film used for fitting are shown in Figure S6. The ferritin spectrum is representative of a biologically sourced ferric (Fe^3+^) phase,^21^ providing iron *L*_2,3_-edge absorption features consistent with a pure ferric phase. The iron *L*_2,3_-edge absorption features of FeCl_2_ are consistent with a pure ferrous (Fe^2+^) phase, which was used as a ferrous standard. The inclusion of a magnetite (Fe_3_O_4_) reference spectrum for fitting was required as this mixed-oxidation state Fe^2+/3+^ phase, previously observed in amyloid structures from AD tissue, provides a unique iron *L*_2,3_-edge absorption spectrum, which is not simply a sum of its principal Fe^2+^ and Fe^3+^ components.^31,62^ The Fe^0^ reference spectrum used for fitting was obtained from Fe^0^ film standards prepared and measured under vacuum to prevent oxidation.^64^ Like magnetite, Fe^0^ was included in the fitting process since we have observed previously Fe^0^ nanodeposits in amyloid plaque structures.^28,59^

Reference spectra were appropriately scaled by normalizing the X-ray absorption intensity for each iron phase to the integrated intensity over the iron *L*_2,3_ absorption edges (Figure S27) as we have described previously.^28,32,59^Figure S28 provides a visualization of the energy ranges used for fitting. An example overlay of an experimental spectrum and its corresponding fit is given in Figure S29.

A table listing the strength of fit for all displayed experimental iron *L*_2,3_-edge absorption spectra is provided in the Supporting Information (Table S1).

## Safety

No new or significant hazards or risks were identified during the reported work.

## Data Availability

The methodology and data sets from this study are available through the Keele University research repository and the Warwick Research Archive Portal.

## Supporting Information Available

Additional STXM data sets from brain tissue sections, methodology, reference iron *L*_2,3_-edge X-ray absorption spectra, and fitting values for the displayed experimental iron *L*_2,3_-edge spectra (PDF)

## Abbreviations

AD: Alzheimer’s disease
ApoE: apolipoprotein E
LCP: left circularly polarized
MRI: magnetic resonance imaging
NFTs: neurofibrillary tangles
ROS: reactive oxygen species
ROI: region of interest
RCP: right circularly polarized
STXM: scanning transmission X-ray microscopy
XRF: X-ray fluorescence
XMCD: X-ray magnetic circular dichroism
