# Supplementary material for: Label-Free In Situ Chemical Characterization of Amyloid Plaques in Human Brain Tissues
Source: ACS Chem Neurosci. 2024 Mar 19;15(7):1469–83. doi: 10.1021/acschemneuro.3c00756 (PMC10995949; doi:10.1021/acschemneuro.3c00756)
Supplement: Supplementary file 1 — cn3c00756_si_001.pdf [file cn3c00756_si_001.pdf]

Supporting Information for:

Label-free *in situ* chemical characterisation of amyloid plaques in human brain tissues

James Everett[1,2]*, Jake Brooks[2], Vindy Tjendana Tjhin[2], Frederik Lermyte[2,3], Ian Hands-Portman[4], Germán Plascencia-Villa[5], George Perry[5], Peter J. Sadler[6], Peter B. O'Connor[6], Joanna F. Collingwood[2] and Neil D. Telling[1]

[1]*School of Pharmacy and Bioengineering, Guy Hilton Research Centre, Thornburrow Drive, Keele University, Staffordshire, ST4 7QB, UK.*

[2]*School of Engineering, Library Road, University of Warwick, Coventry, CV4 7AL, UK.*

[3]*Department of Chemistry, Technical University of Darmstadt, Alarich-Weiss-Strasse 4, 64287 Darmstadt, Germany.*

[4]*School of Life Sciences, Gibbet Hill Campus, University of Warwick, Coventry, CV4 7AL, UK.*

[5]*Department of Developmental and Regenerative Biology. The University of Texas at San Antonio (UTSA), San Antonio, TX, 78249, USA.*

[6]*Department of Chemistry, Library Road, University of Warwick, Coventry CV4 7AL, UK.*

***Correspondence to:** j.everett@keele.ac.uk

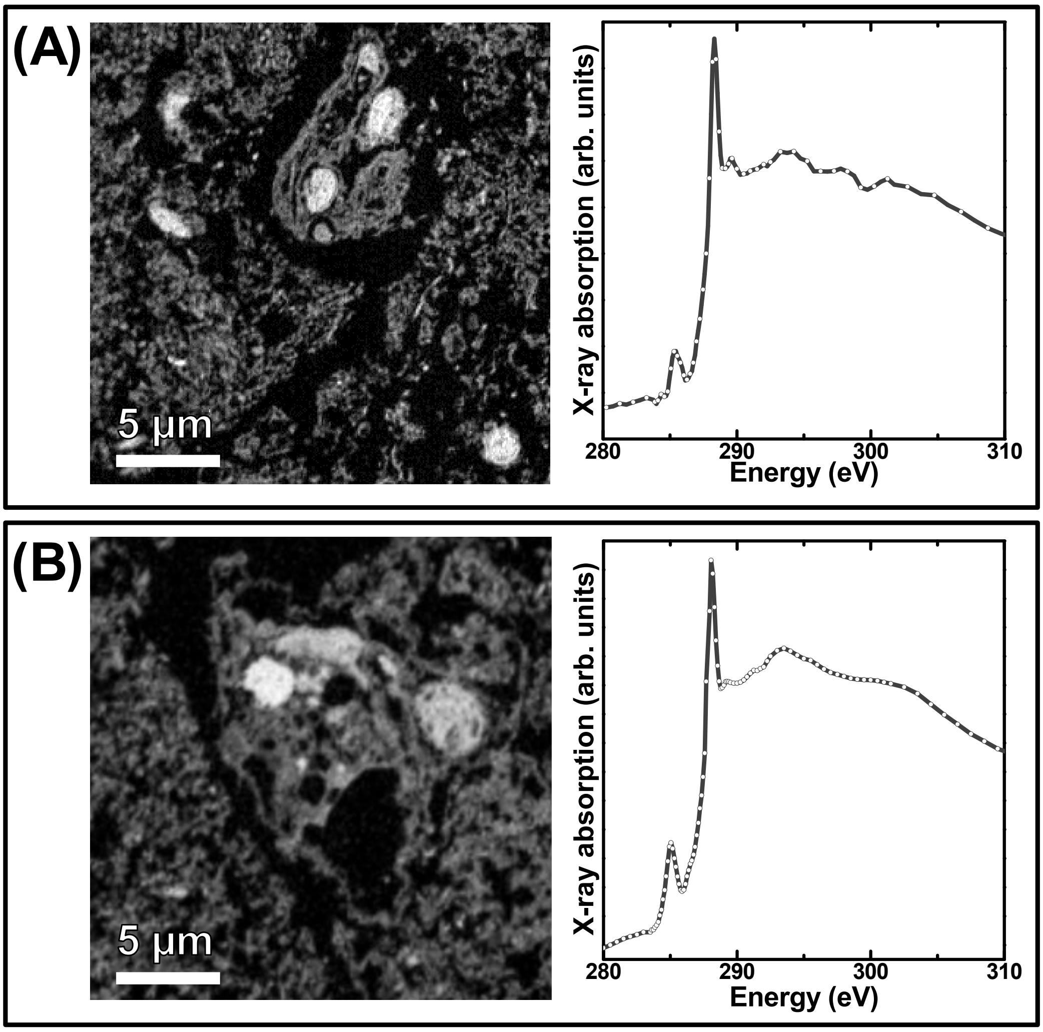

Fig. S1 Carbon *K*-edge amide speciation maps (images) and associated x-ray absorption spectra from cellular material within a 200 nm thick section of AD hippocampus. The 287.5 eV C-H absorption feature observed in equivalent spectra from amyloid plaques was not apparent in these cellular regions.

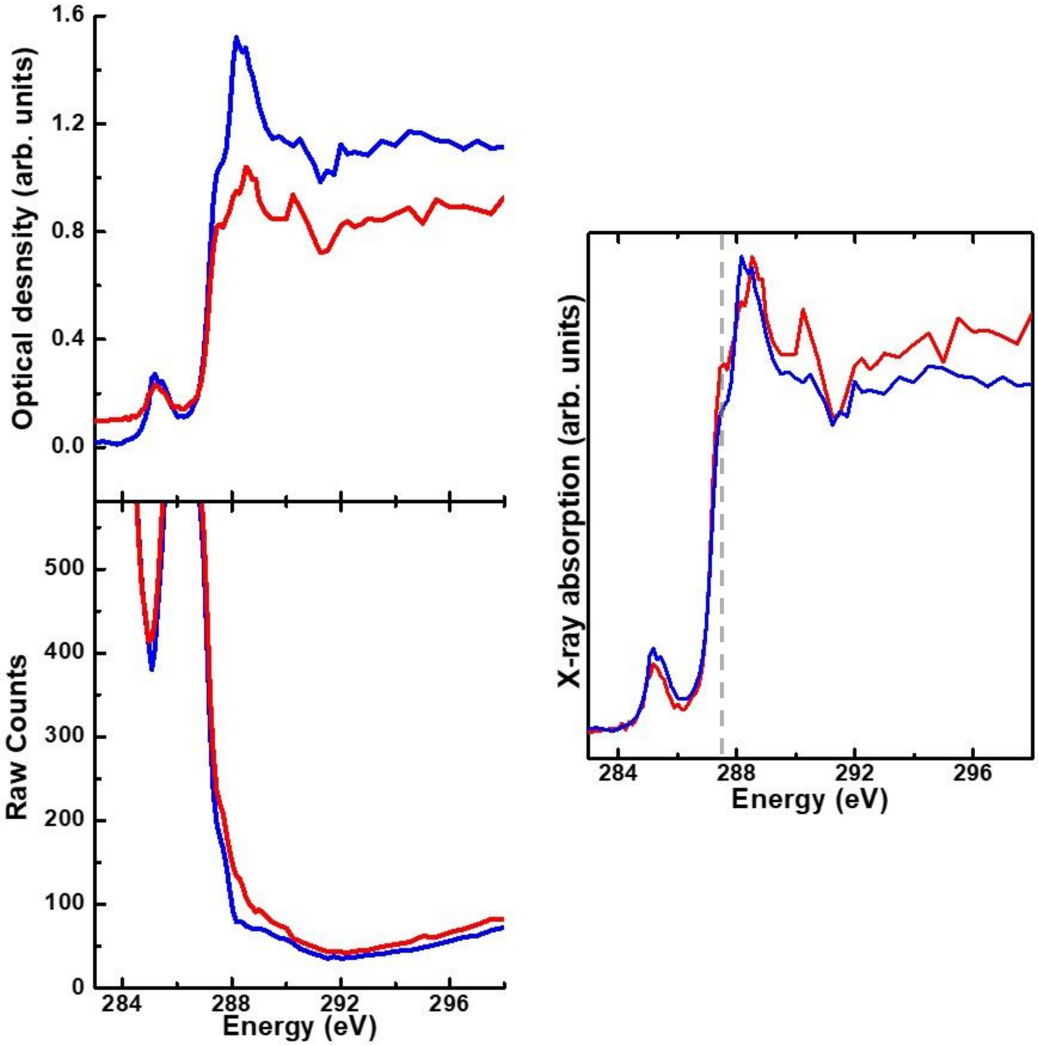

Fig. S2 Comparison of carbon *K*-edge x-ray absorption spectra from two differing regions of the isolated plaque core in Fig. 3 of the main text. The left hand panels show that both regions (i) strongly absorb x-rays at 287.5 eV regardless of differing optical density, (ii) do not reach saturation limits. The right hand panel shows that, despite being from a less optically dense region, the 287.5 eV feature (dotted line) is more prominent in red trace (compared to the blue trace), where spectra are scaled to carbon *K*-edge maxima.

STXM analysis of isolated plaque cores

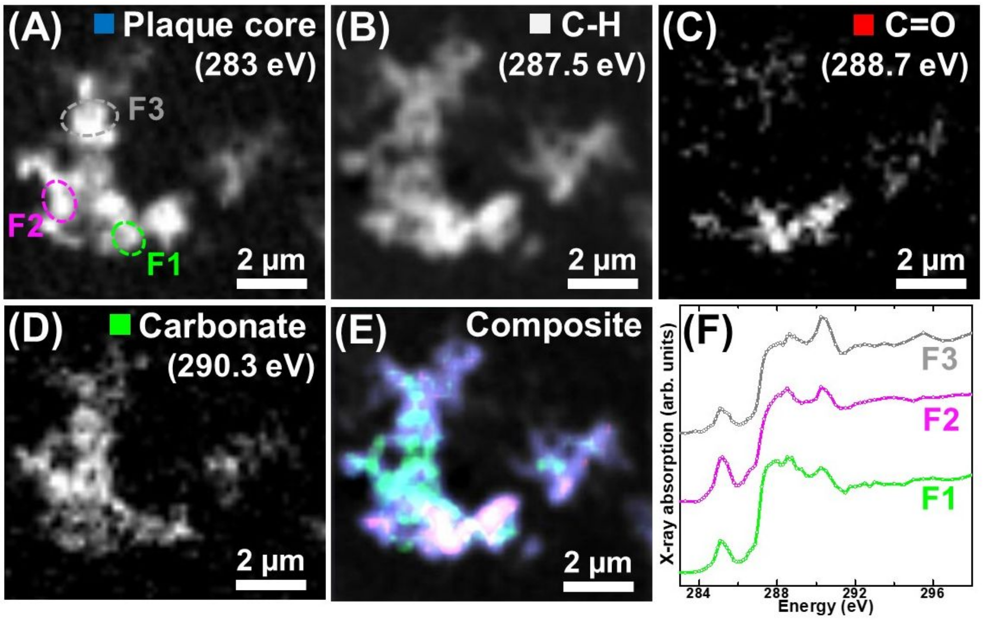

Fig. S3 Carbon *K*-edge x-ray images, speciation maps and x-ray absorption spectra of an isolated amyloid plaque core from subject X. **(A)** Single energy 283 eV image showing overall plaque morphology. **(B)** C-H map. **(C)** C=O map. **(D)** Carbonate map. **(H)** Composite image showing the morphology (blue), C-H (grey), C=O (red) and carbonate (green) content of the plaque. **(F)** Carbon *K*-edge x-ray absorption spectra from the plaque areas highlighted in panel (A). This plaque was originally presented in Figs. 3 and 5 of Everett et al. *Nanoscale*, 2018 (1).

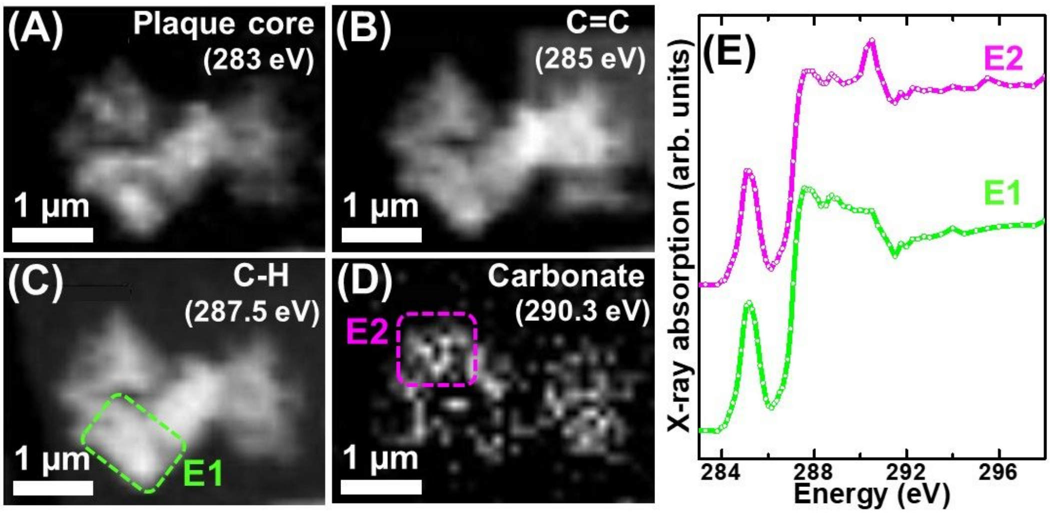

Fig. S4 Carbon *K*-edge x-ray image, speciation maps and x-ray absorption spectra of an isolated amyloid plaque core from subject X. **(A)** Single energy 283 eV image showing overall plaque morphology. **(B)** C=C map. **(C)** C-H map. **(D)** Carbonate map. **(E)** Carbon *K*-edge x-ray absorption spectra from the plaque areas highlighted in panels (C) and (D).

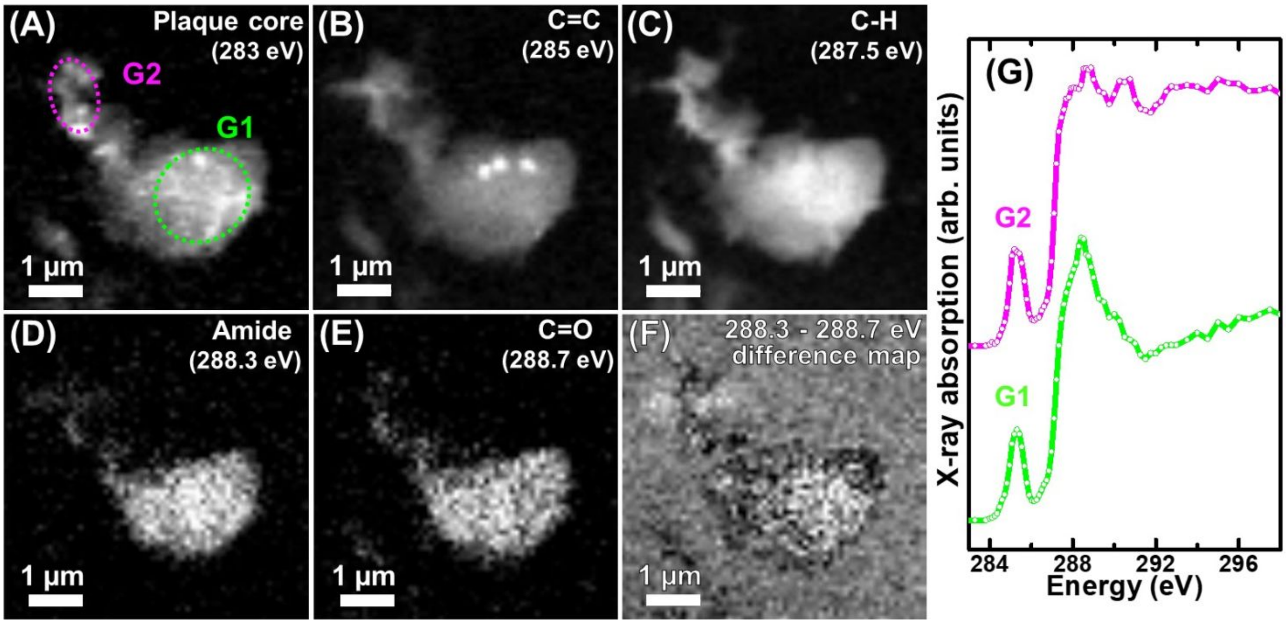

Fig. S5 Carbon *K*-edge x-ray images, speciation maps and x-ray absorption spectra of an isolated amyloid plaque core from subject Y. **(A)** Single energy 283 eV image showing overall plaque morphology. **(B)** C=C map. **(C)** C-H map. **(D)** Amide map. **(E)** C=O map. **(F)** Carbon chemistry difference map, where areas of light contrast strongly absorb at amide feature and areas of dark contrast absorb strongly simple carbonyl feature. **(G)** Carbon *K*-edge x-ray absorption spectra from the plaque areas highlighted in panel (A). This plaque was originally presented in Fig. 4 of Everett et al. *Nanoscale*, 2018 (1).

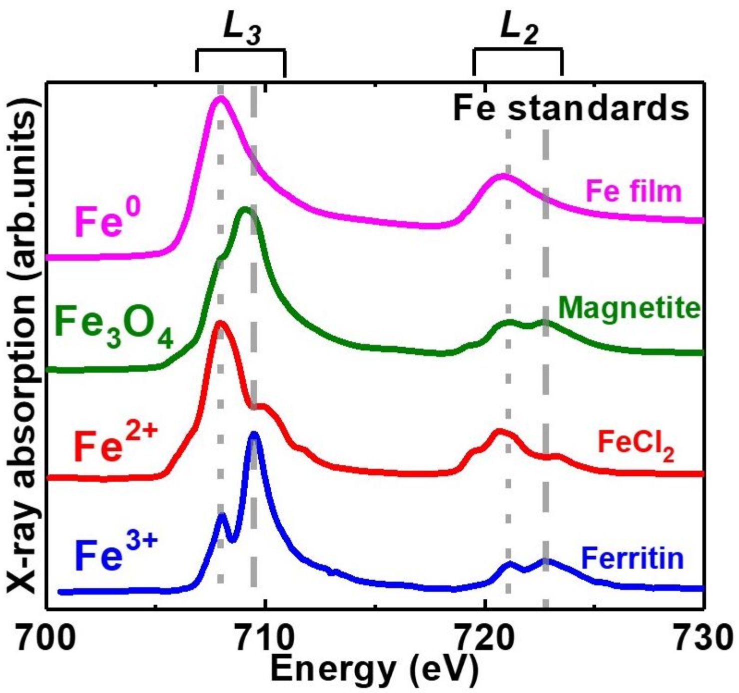

Fig. S6 Reference iron $L_{2,3}$-edge x-ray absorption spectra from four iron standards. Dotted and dashed lines show the principal energy positions for Fe^{2+}/Fe^0 and Fe^{3+} absorption peaks respectively. Ferric (Fe^{3+}) materials (blue spectrum) display principal x-ray absorption features at 709.5 and 723 eV, whereas ferrous (Fe^{2+}; red spectrum) and zero–oxidation-state metallic (Fe^0; magenta spectrum) phases display features at 708 and 721 eV. Although Fe^{2+} and Fe^0 share absorption features at identical energies, Fe^0 can be further distinguished from Fe^{2+} by its broadened absorption peaks and enhanced L_2/L_3 peak ratios. A shoulder feature on the principal Fe^{3+} cation feature is characteristic of the mixed-valence mineral magnetite (green spectrum).

STXM analysis of AD tissue section plaques

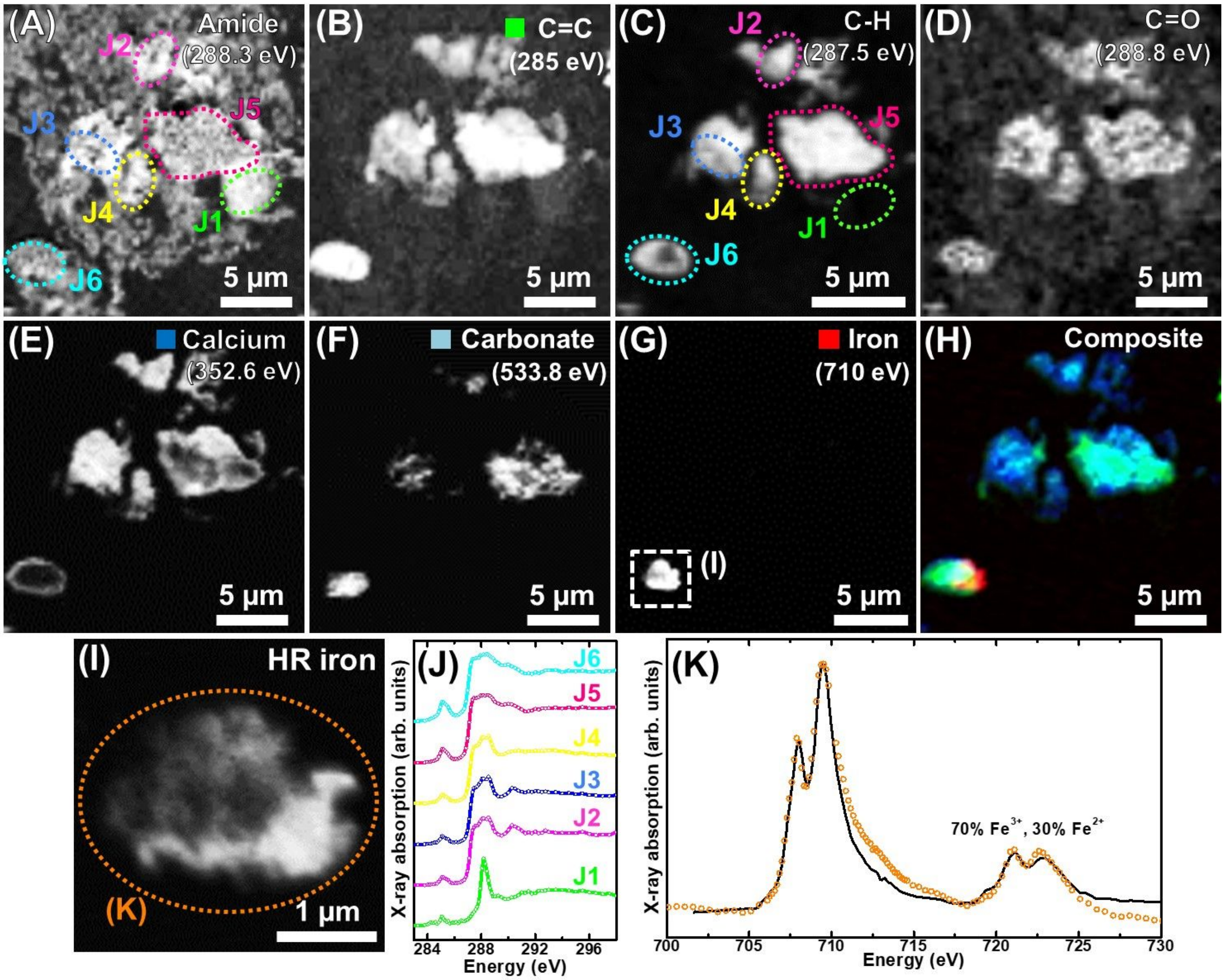

Fig. S7 STXM examination of an amyloid plaque region located within a 200 nm thick AD amygdala section. (A-D) Carbon *K*-edge speciation maps. **(A)** Amide map showing overall tissue structure for the region. **(B)** C=C map, **(C)** C-H map, **(D)** C=O map. **(E)** Calcium *L*-edge map. **(F)** Oxygen *K*-edge carbonate map. **(G)** Iron L_3-edge map. **(H)** Composite map showing C=C (green), calcium (blue), carbonate (sky blue) and iron (red) content. **(I)** High resolution iron L_3-edge map of the region highlighted in (G). **(J)** Carbon *K*-edge x-ray absorption spectra from the plaque areas highlighted in panels (A) and (C). **(K)** Iron $L_{2,3}$-edge x-ray absorption spectrum from the area highlighted in (I). This tissue region was originally presented in Figure 2 of Everett et al. *Microscopy and Microanalysis*, 2018 (2).

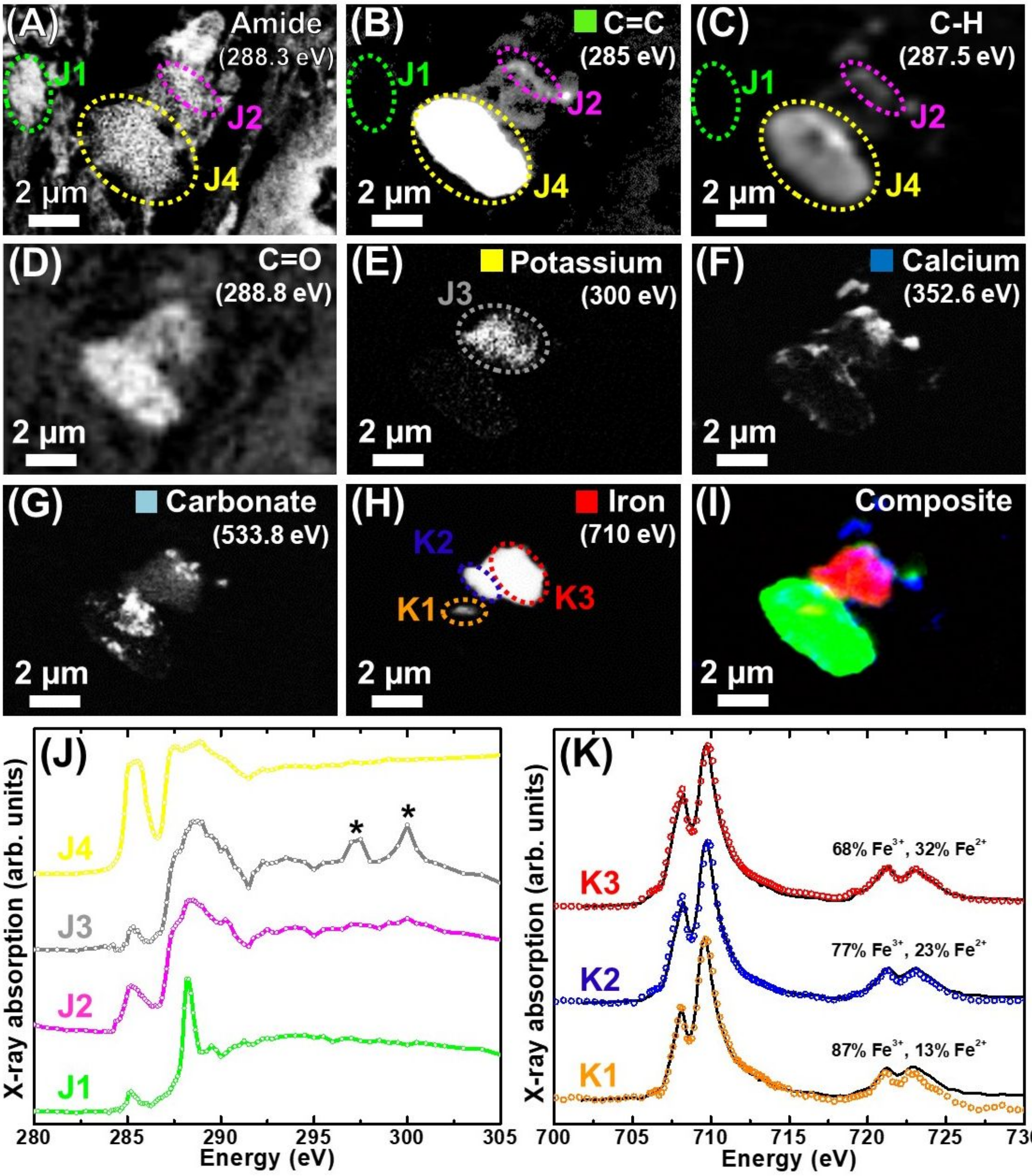

Fig. S8 STXM examination of an amyloid plaque located within a 200 nm thick AD amygdala section. (A-D) Carbon *K*-edge speciation maps. **(A)** Amide map, **(B)** C=C map, **(C)** C-H map, **(D)** C=O map. **(E)** Potassium *L*-edge map. **(F)** Calcium *L*-edge map. **(G)** Oxygen *K*-edge carbonate map. **(H)** Iron L_3-edge map. **(I)** Composite map showing C=C (green), calcium (blue), carbonate (sky blue) and iron (red) content. **(J)** Carbon *K*-edge x-ray absorption spectra from the plaque areas highlighted in panels (A-C) and (D). The asterisks in spectrum J3 denote potassium absorption features. **(K)** Iron $L_{2,3}$-edge x-ray absorption spectra from the plaque areas highlighted in (H).

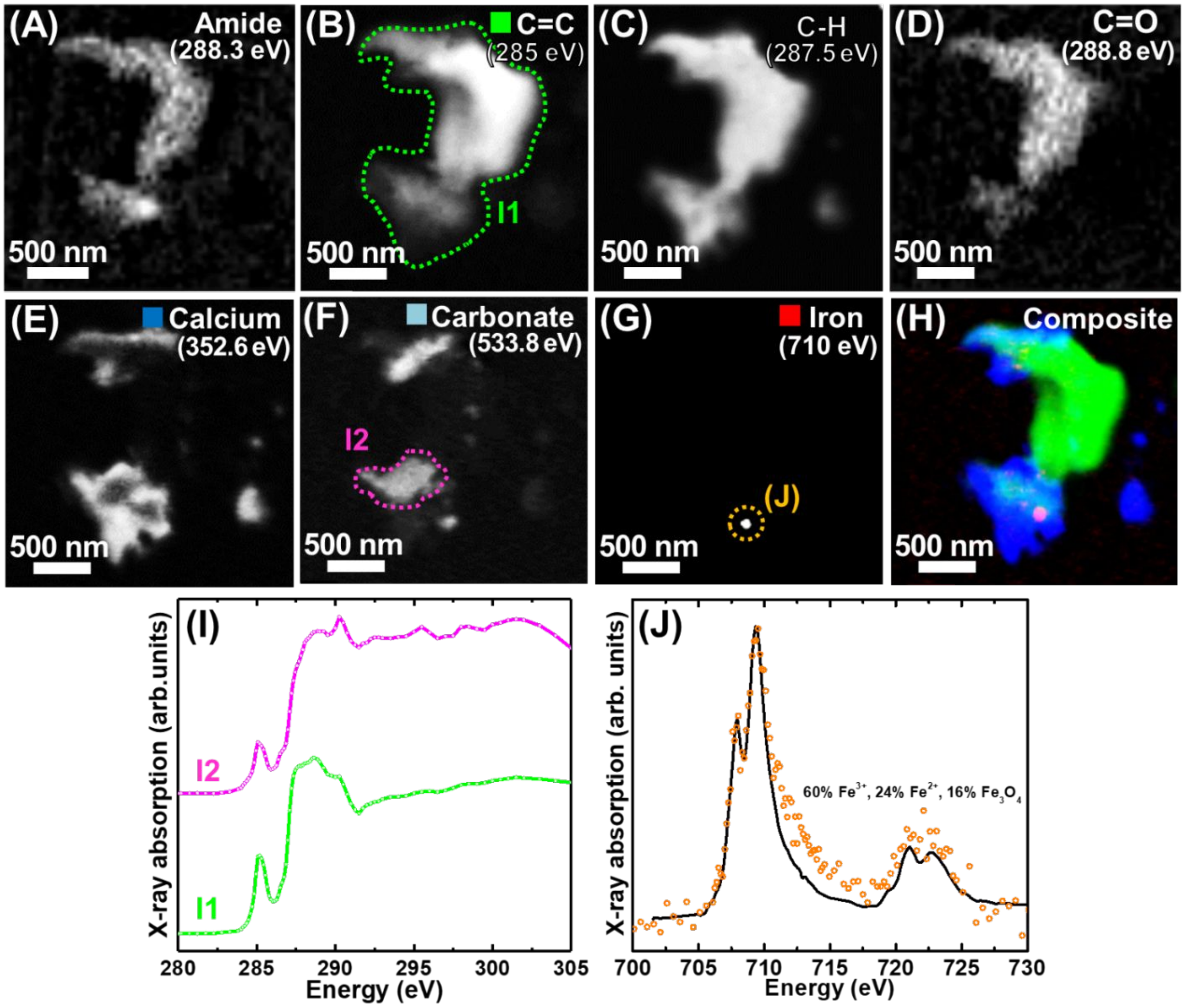

Fig. S9 STXM examination of an amyloid plaque located within a 200 nm thick AD amygdala section. (A-D) Carbon *K*-edge speciation maps. **(A)** Amide map, **(B)** C=C map, **(C)** C-H map, **(D)** C=O map. **(E)** Calcium *L*-edge map. **(F)** Oxygen *K*-edge carbonate map. **(G)** Iron L_3-edge map. **(H)** Composite map showing C=C (green), calcium (blue), carbonate (sky blue) and iron (red) content. **(I)** Carbon *K*-edge x-ray absorption spectra from the plaque areas highlighted in panels (B) and (F). **(J)** Iron $L_{2,3}$-edge x-ray absorption spectrum from the plaque area highlighted in (G).

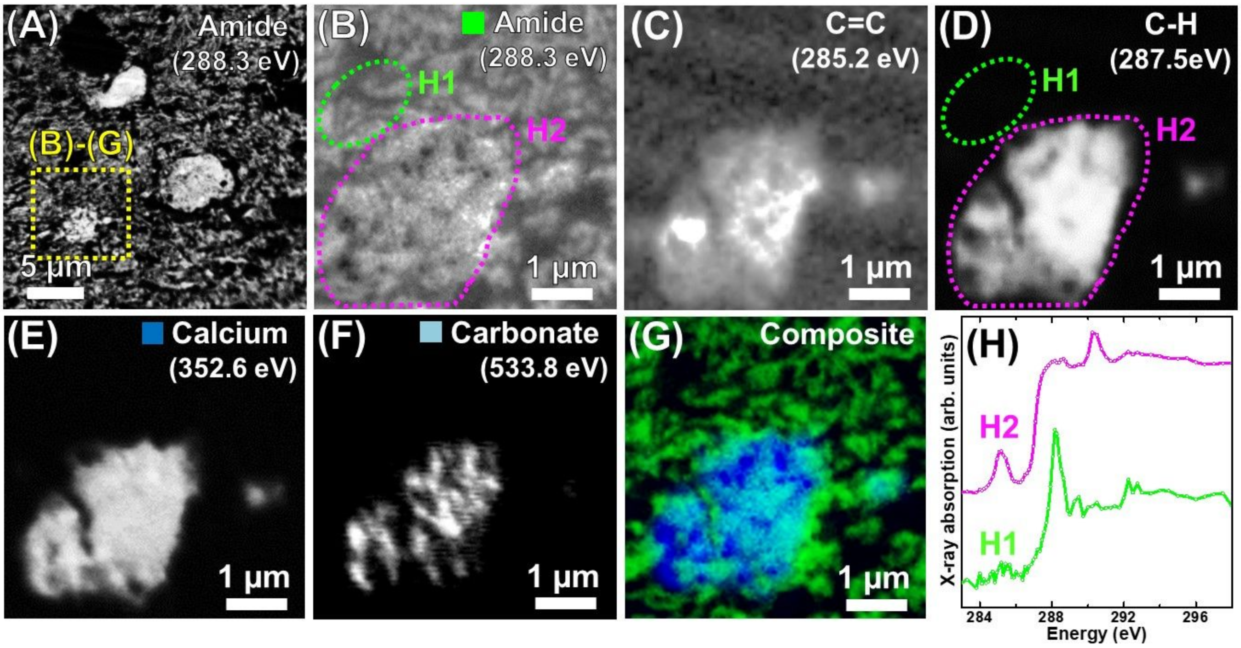

Fig. S10 STXM examination of an amyloid plaque located within a 200 nm thick AD amygdala section. **(A)** Amide overview map showing tissue structure for the region containing the plaque. The plaque is located within the yellow box. (B-D) Carbon *K*-edge speciation maps of the plaque. **(B)** Amide map, **(C)** C=C map, **(D)** C-H map. **(E)** Calcium *L*-edge map. **(F)** Oxygen *K*-edge carbonate map. **(G)** Composite map showing amide (green), calcium (blue), carbonate (sky blue) content. **(H)** Carbon *K*-edge x-ray absorption spectra from the plaque areas highlighted in panels (B) and (D).

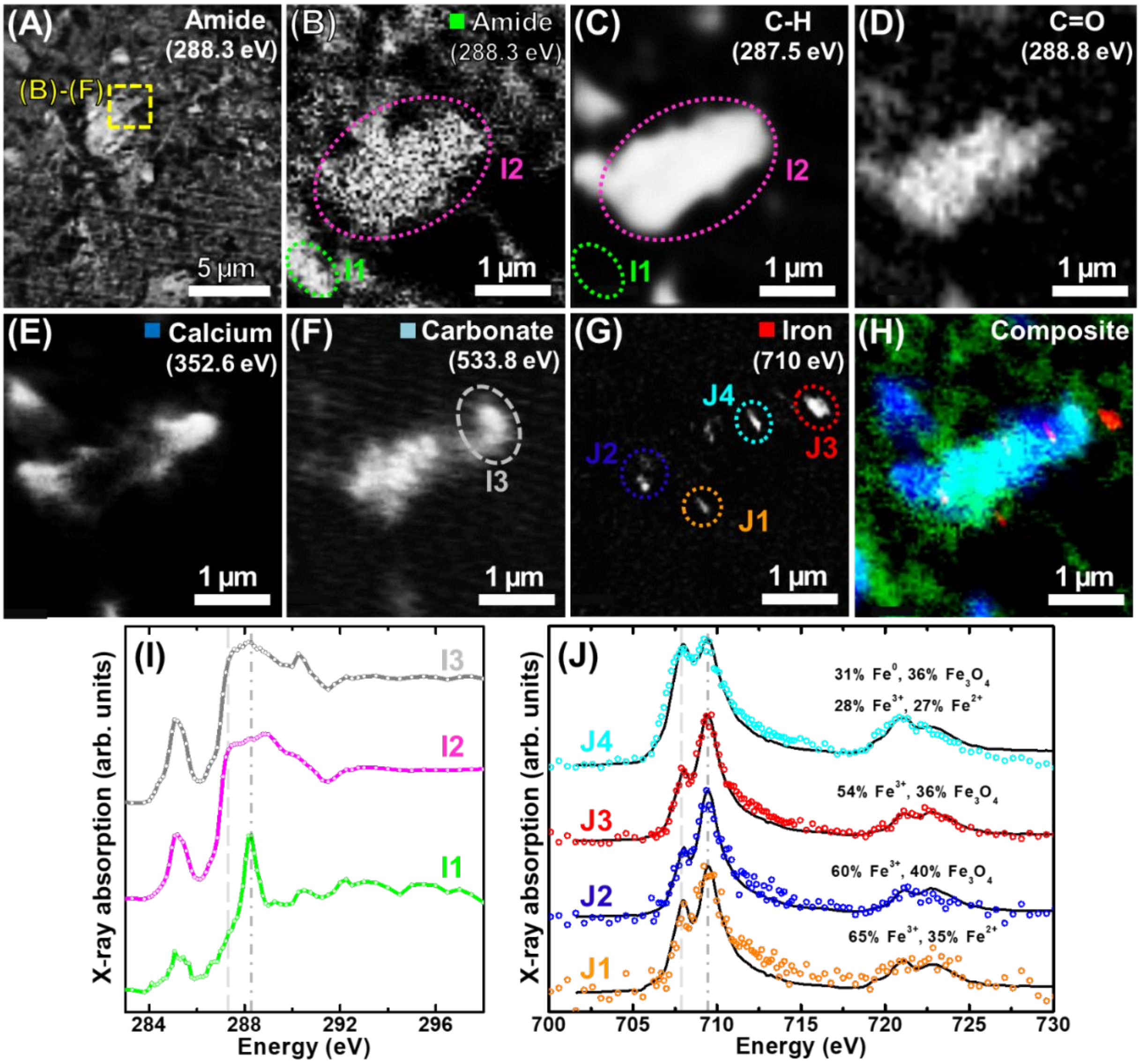

Fig. S11 STXM examination of an amyloid plaque located within a 200 nm thick AD amygdala section. **(A)** Amide overview map showing tissue structure for the region containing the plaque. The plaque is highlighted by the yellow box. (B-D) Carbon *K*-edge speciation maps of the plaque. **(B)** Amide map, **(C)** C-H map, **(D)** C=O map. **(E)** Calcium *L*-edge map. **(F)** Oxygen *K*-edge carbonate map. **(G)** Iron L_3-edge map. **(H)** Composite map showing amide (green), calcium (blue), carbonate (sky blue) and iron (red) content. **(I)** Carbon *K*-edge x-ray absorption spectra from the plaque areas highlighted in panels (B), (C) and (F). The energies corresponding to the C-H (287.5 eV) and amide (288.3 eV) absorption features are shown by the dashed and dotted-dashed lines, respectively. **(J)** Iron $L_{2,3}$-edge x-ray absorption spectra from the areas highlighted in (G). Dashed and dotted-dashed lines show the principal energy positions for Fe^{2+}/Fe^{0} and Fe^{3+} absorption peaks, respectively.

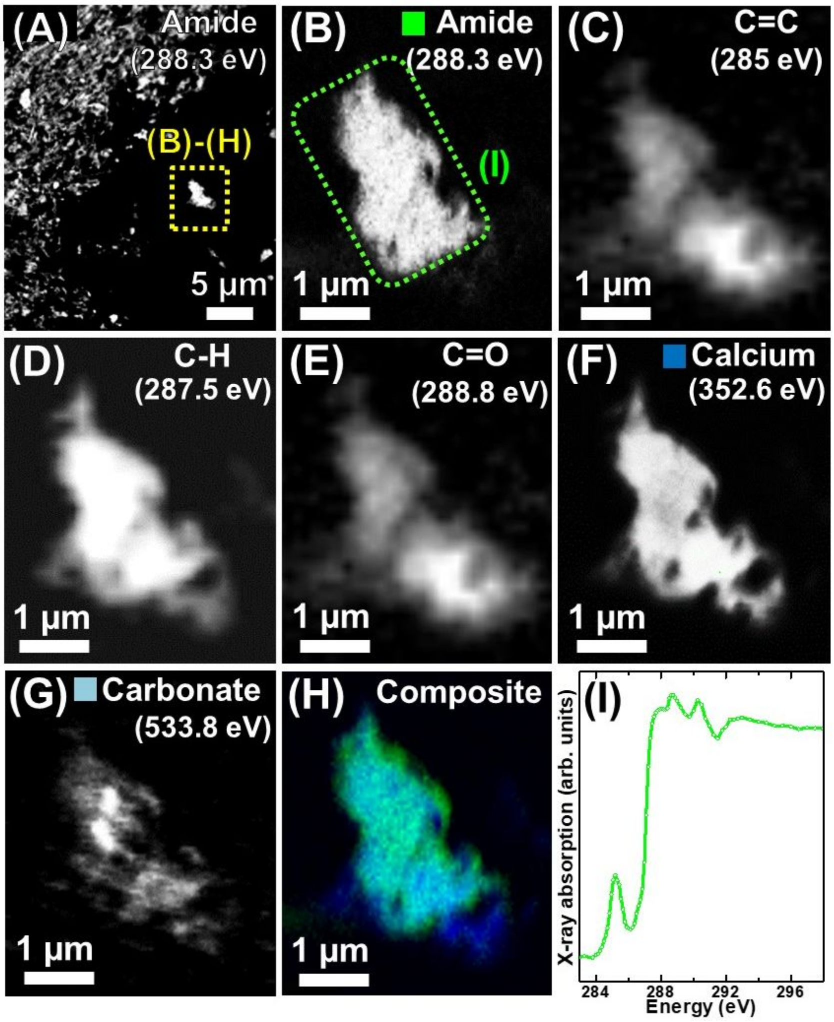

Fig. S12 STXM examination of an amyloid plaque located within a 200 nm thick AD amygdala section. **(A)** Amide overview map showing tissue structure for the region containing the plaque. The plaque is highlighted by the yellow box. (B-E) Carbon *K*-edge speciation maps of the plaque. **(B)** Amide map, **(C)** C=C map, **(D)** C-H map, **(E)** C=O map. **(F)** Calcium *L*-edge map. **(G)** Oxygen *K*-edge carbonate map. **(H)** Composite map showing amide (green), calcium (blue), carbonate (sky blue) content. **(I)** Carbon *K*-edge x-ray absorption spectra from the plaque as highlighted in panel (B).

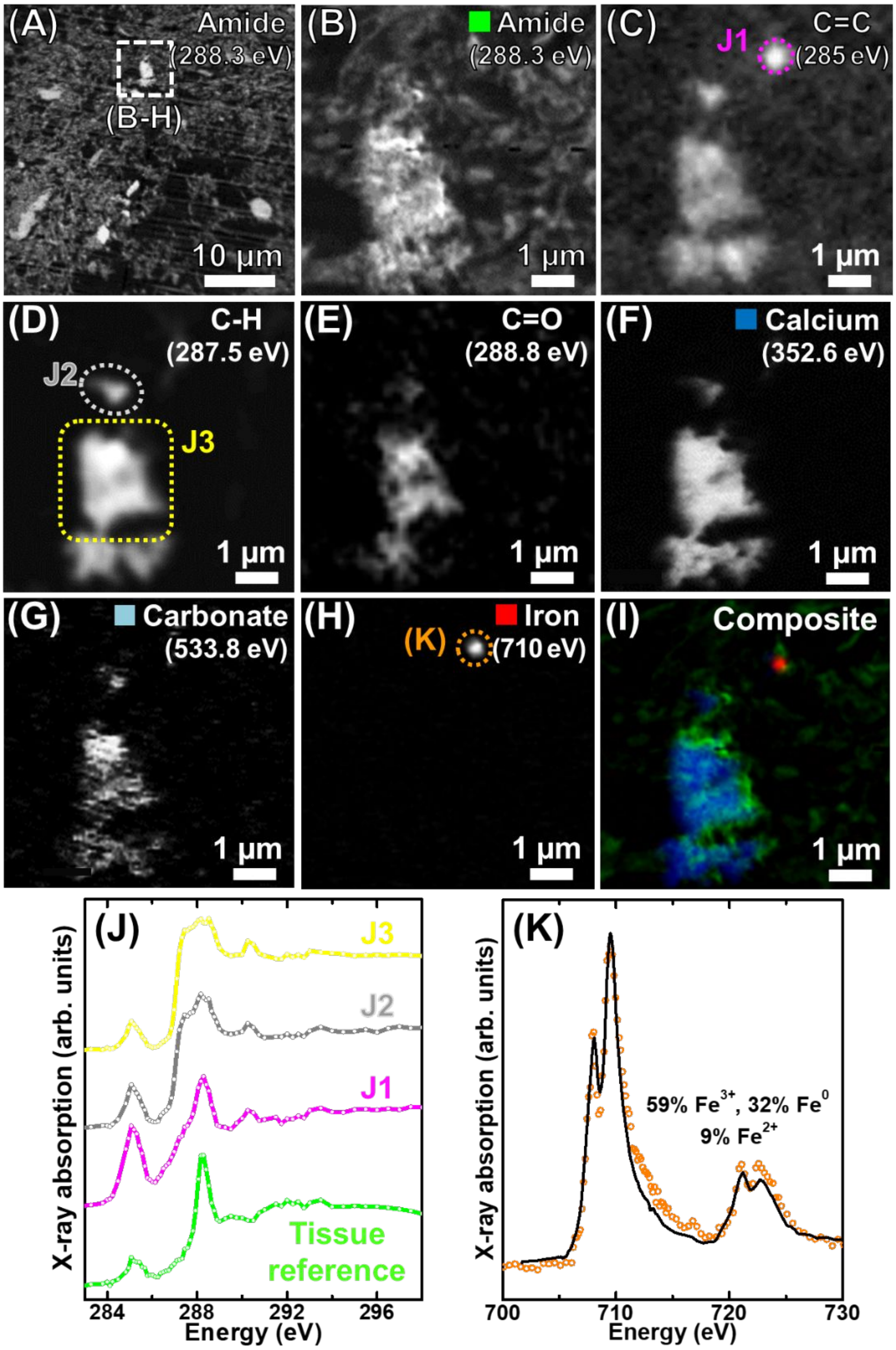

Fig. S13 STXM examination of an amyloid plaque located within a 200 nm thick AD amygdala section. **(A)** Amide overview map showing tissue structure for the region containing the plaque. The plaque is highlighted by the white box. (B-E) Carbon *K*-edge speciation maps of the plaque. **(B)** Amide map, **(C)** C=C map, **(D)** C-H map, **(E)** C=O map. **(F)** Calcium *L*-edge map. **(G)** Oxygen *K*-edge carbonate map. **(H)** Iron L_3-edge map. **(I)** Composite map showing amide (green), calcium (blue), carbonate (sky blue) and iron (red) content. **(J)** Carbon *K*-edge x-ray absorption spectra from the plaque areas highlighted in panels (C) and (D). The tissue reference spectrum (green trace) is derived from the neuropil surrounding the plaque. **(K)** Iron $L_{2,3}$-edge x-ray absorption spectrum from the iron deposit highlighted in (H).

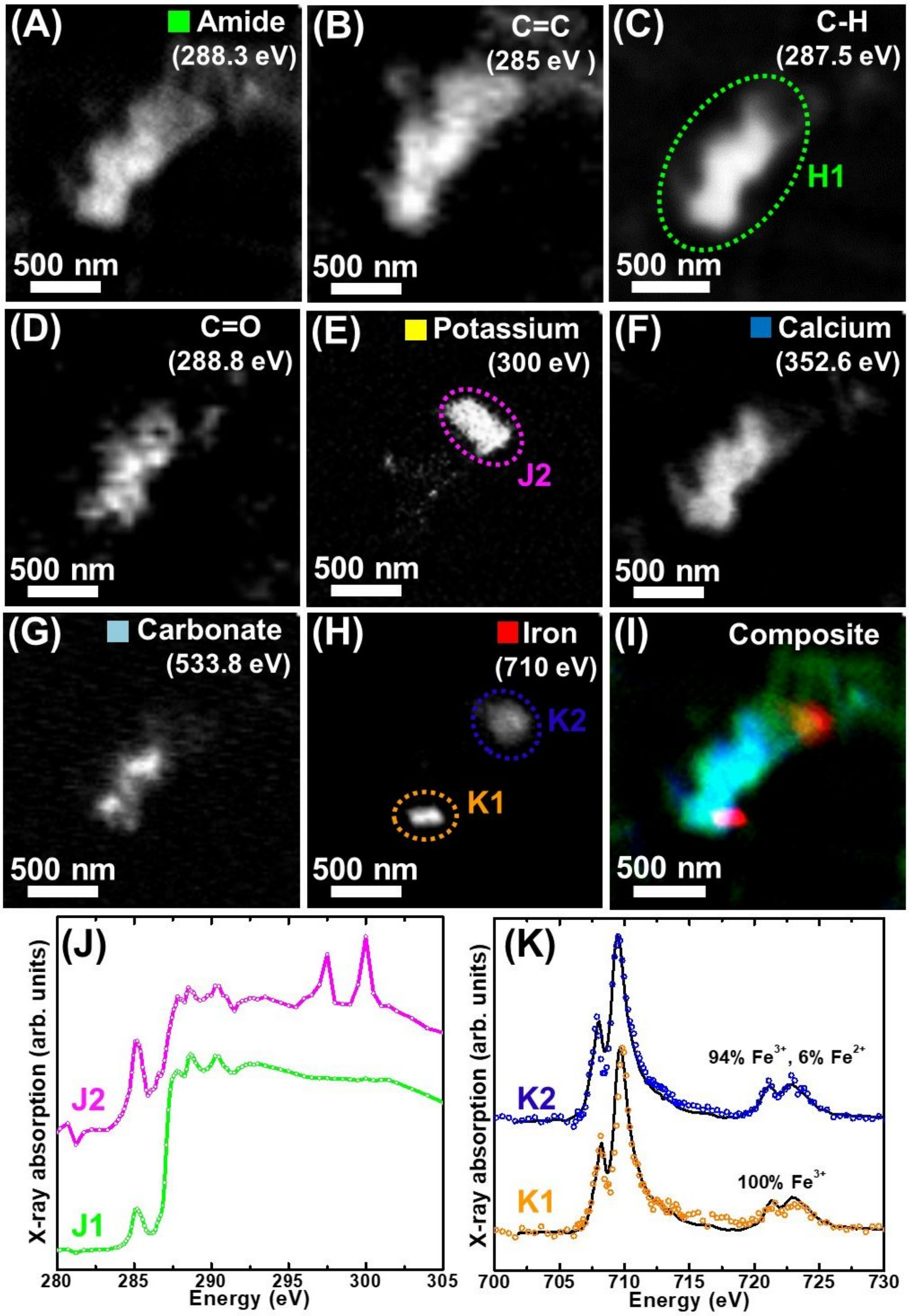

Fig. S14 STXM examination of amyloid plaque located within a 200 nm thick AD amygdala section. (A-D) Carbon *K*-edge speciation maps. **(A)** Amide map, **(B)** C=C map, **(C)** C-H map, **(D)** C=O map. **(E)** Potassium *L*-edge map. **(F)** Calcium *L*-edge map. **(G)** Oxygen *K*-edge carbonate map. **(H)** Iron L_3-edge map. **(I)** Composite map showing amide (green), calcium (blue), carbonate (sky blue) and iron (red) content. **(J)** Carbon *K*-edge x-ray absorption spectra from the plaque areas highlighted in panels (C) and (E). **(K)** Iron $L_{2,3}$-edge x-ray absorption spectra from the plaque areas highlighted in (H).

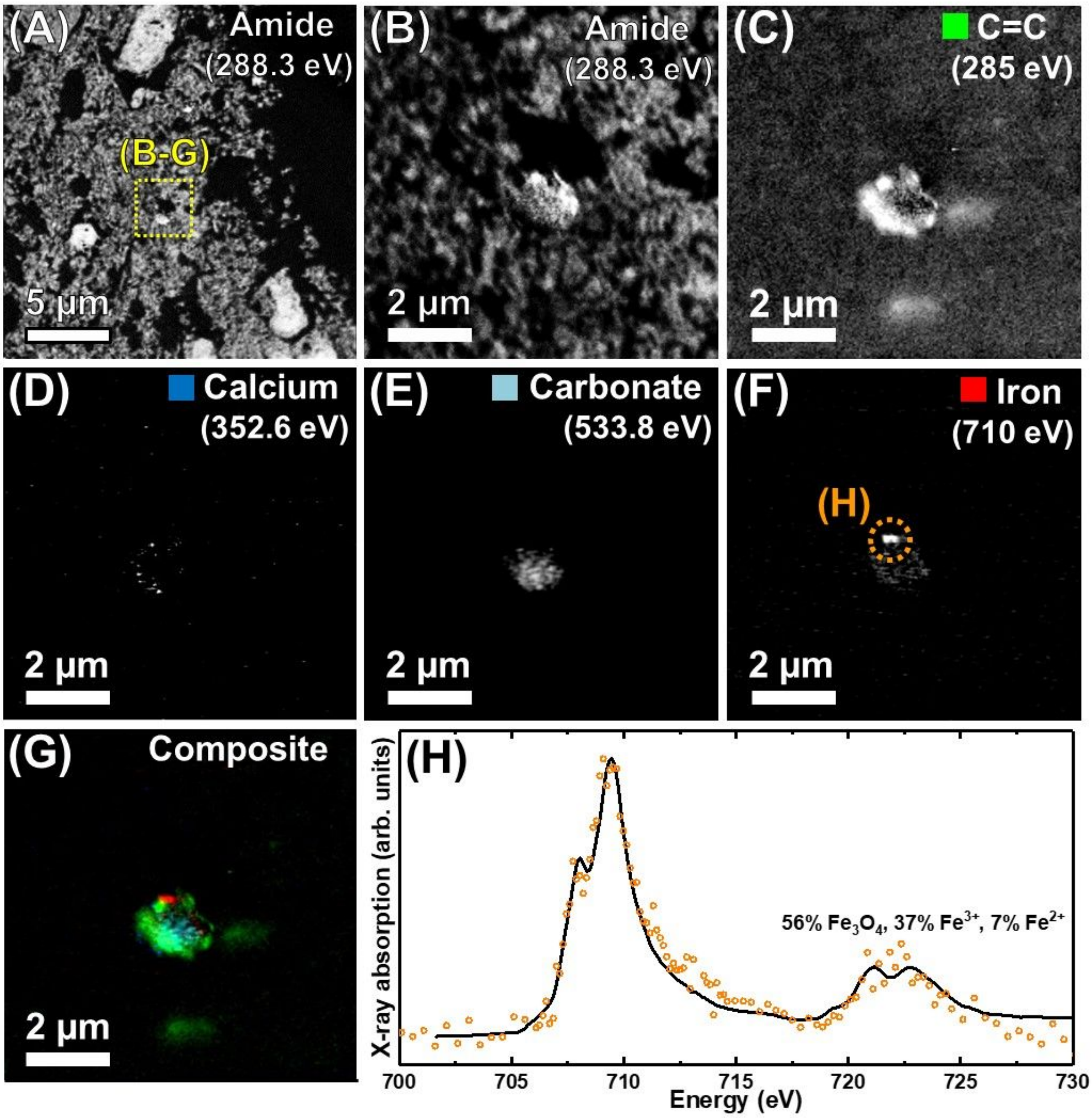

Fig. S15 STXM examination of an amyloid plaque located within a 200 nm thick AD amygdala section. **(A)** Amide overview map showing tissue structure for the region containing the plaque. The plaque is highlighted by the yellow box. (B-G) Speciation maps of the plaque. **(B)** Amide map. **(C)** C=C map. **(D)** Calcium *L*-edge map. **(E)** Oxygen *K*-edge carbonate map. **(F)** Iron L_3-edge map. **(G)** Composite map showing C=C (green), calcium (blue), carbonate (sky blue) and iron (red) content. **(H)** Iron $L_{2,3}$-edge x-ray absorption spectrum from the iron deposit highlighted in (F).

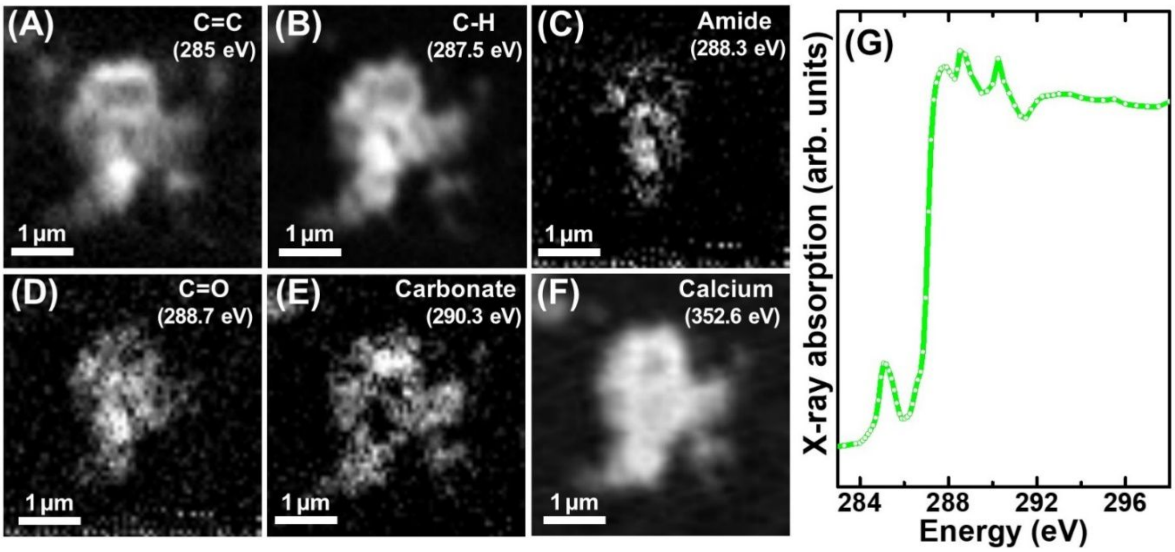

Fig. S16 STXM examination of an amyloid plaque located within a 200 nm thick AD amygdala section. **(A)** C=C map **(B)** C-H map. **(C)** Amide map. **(D)** C=O map. **(E)** Carbon *K*-edge carbonate map. **(F)** Calcium *L*-edge map. **(G)** Carbon *K*-edge x-ray absorption spectrum from the plaque.

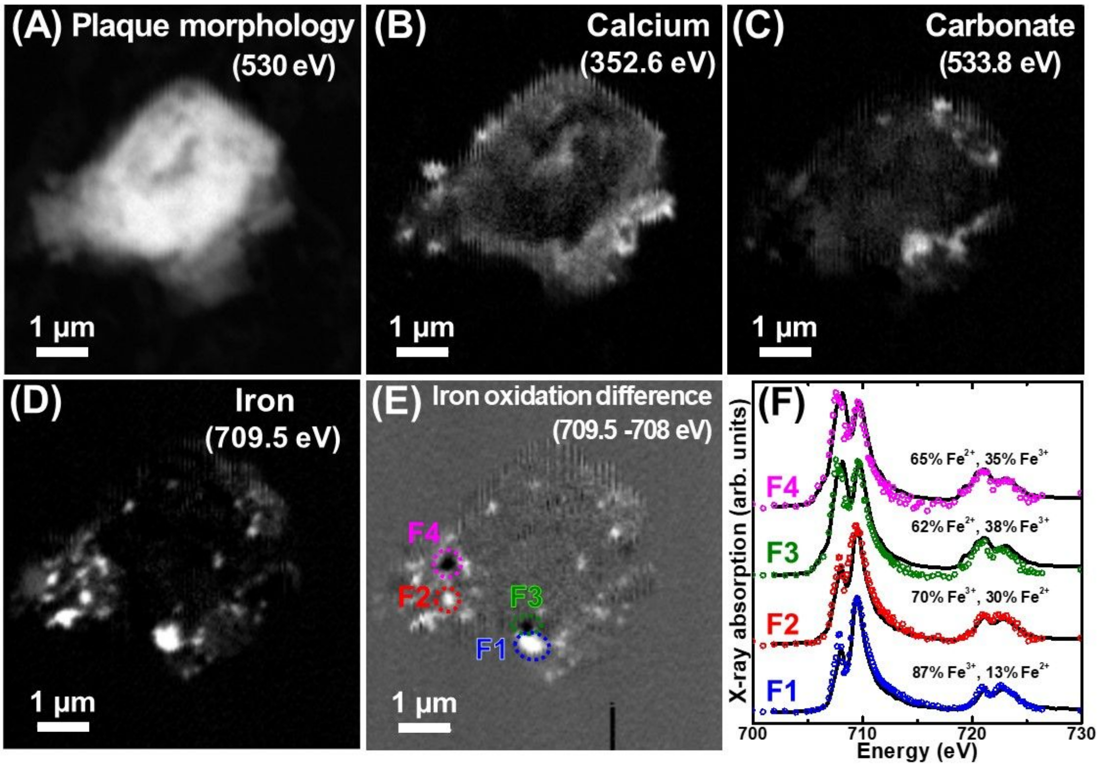

Fig. S17 STXM examination of an amyloid plaque located within a 200 nm thick AD hippocampal section. **(A)** Single 530 eV image showing plaque morphology. **(B)** Calcium *L*-edge map. **(C)** Oxygen *K*-edge carbonate map. **(D)** Iron L_3-edge map. **(E)** Iron L_3-edge oxidation state difference map showing strongly absorbing oxidized iron (Fe^{3+}) as light contrast, and chemically reduced iron (Fe^{2+} and/or Fe^0) as dark contrast. **(F)** Iron $L_{2,3}$-edge x-ray absorption spectra from the plaque areas highlighted in (E).

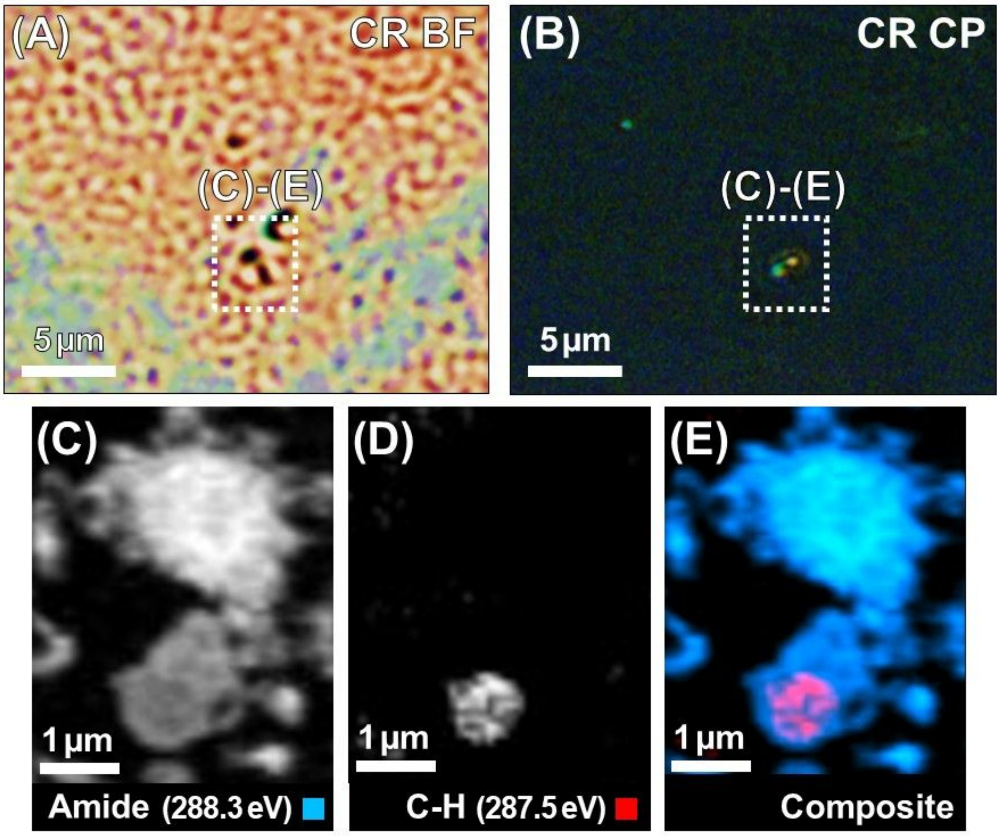

Fig. S18 Correlative Congo red and STXM examination of an amyloid plaque within adjacent AD hippocampal sections. **(A)** Brightfield image of a tissue region stained with Congo red. **(B)** The tissue region shown in (A) imaged using cross polarized light. Structures can be seen to display "apple green" birefringence characteristic of amyloid. (C)-(E) STXM images of the amyloid containing tissue region highlighted in (A) and (B) in an adjacent unstained tissue section. **(C)** Amide speciation map. **(D)** C-H speciation map. **(E)** Composite image showing amide (blue) and C-H (red) distribution.

STXM analysis of control tissue section plaques

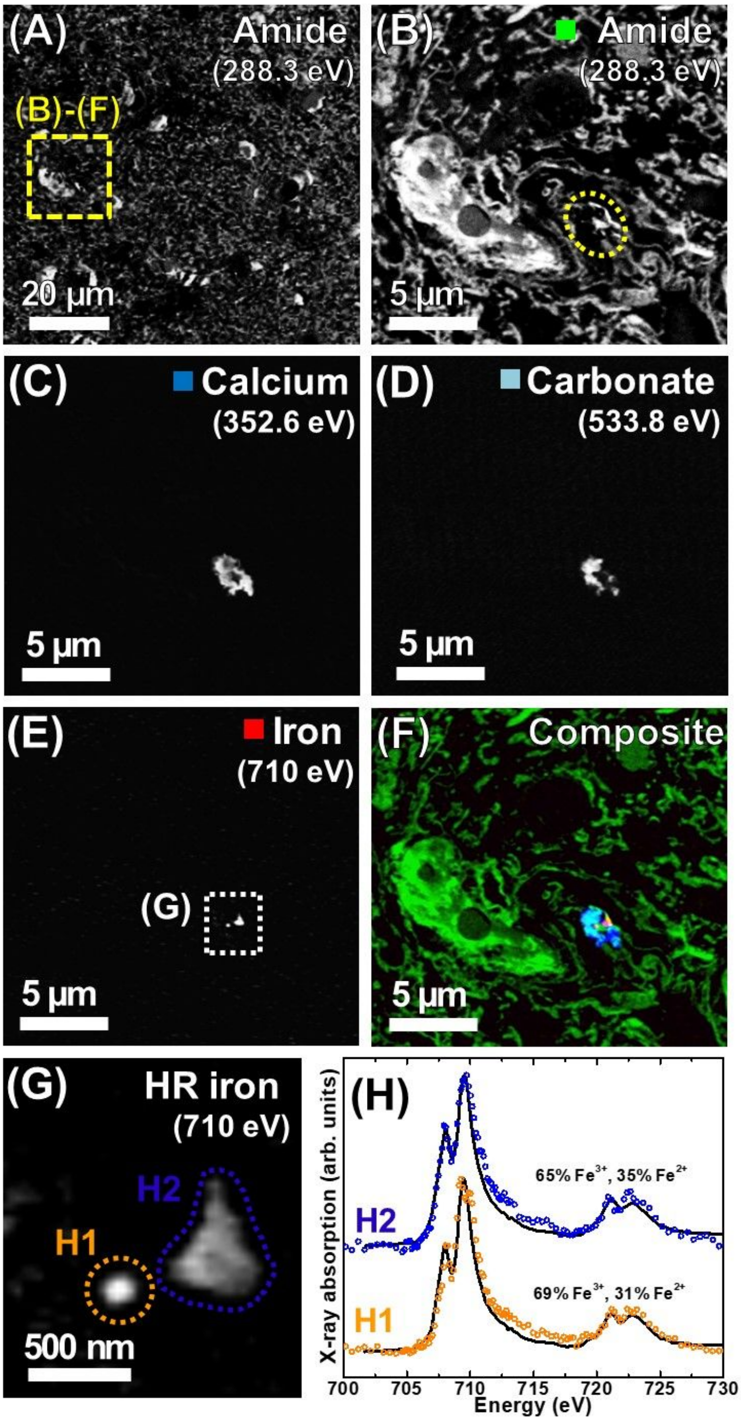

Fig. S19 STXM examination of an amyloid plaque located within a 200 nm thick amygdala section from a neurologically healthy control. **(A)** Amide overview map. (B-G) STXM speciation maps of the plaque-containing tissue region. **(B)** Amide map. The yellow highlighted area is a suspected amyloid plaque. **(C)** Calcium *L*-edge map. **(D)** Oxygen *K*-edge carbonate map. **(E)** Iron L_3-edge map. **(F)** Composite image showing amide (green), calcium (blue), carbonate (sky blue) and iron (red) content. **(G)** High resolution iron L_3-edge map of the plaque area highlighted in (E). **(H)** Iron $L_{2,3}$-edge x-ray absorption spectra from the iron deposits highlighted in (G).

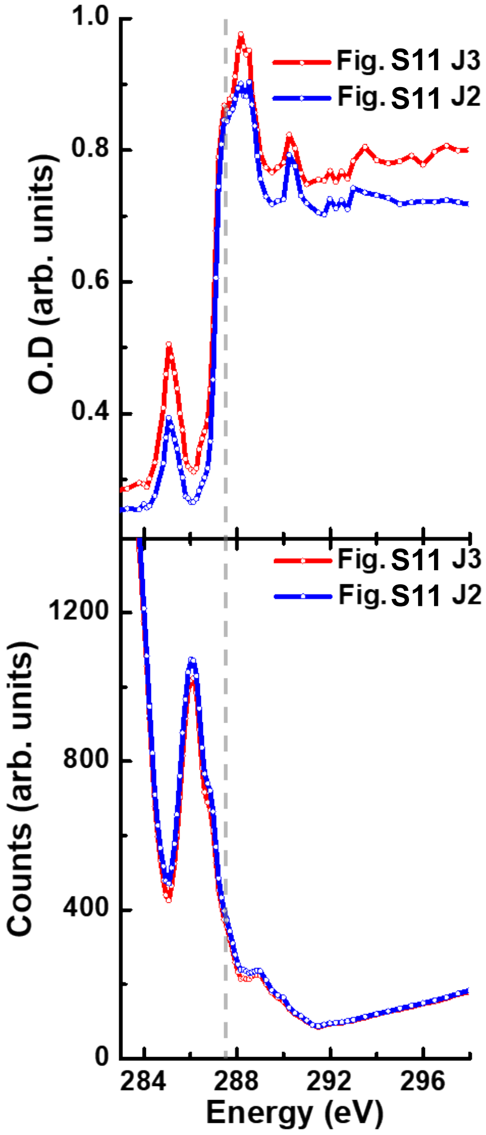

Fig. S20 Comparison of 287.5 eV C-H absorption feature in carbon *K*-edge spectra (highlighted by the dotted line) from differing regions of the suspected plaque within presented in Fig. S11. The feature is prominent across multiple plaque regions of differing optical density, which did not reach the saturation limit.

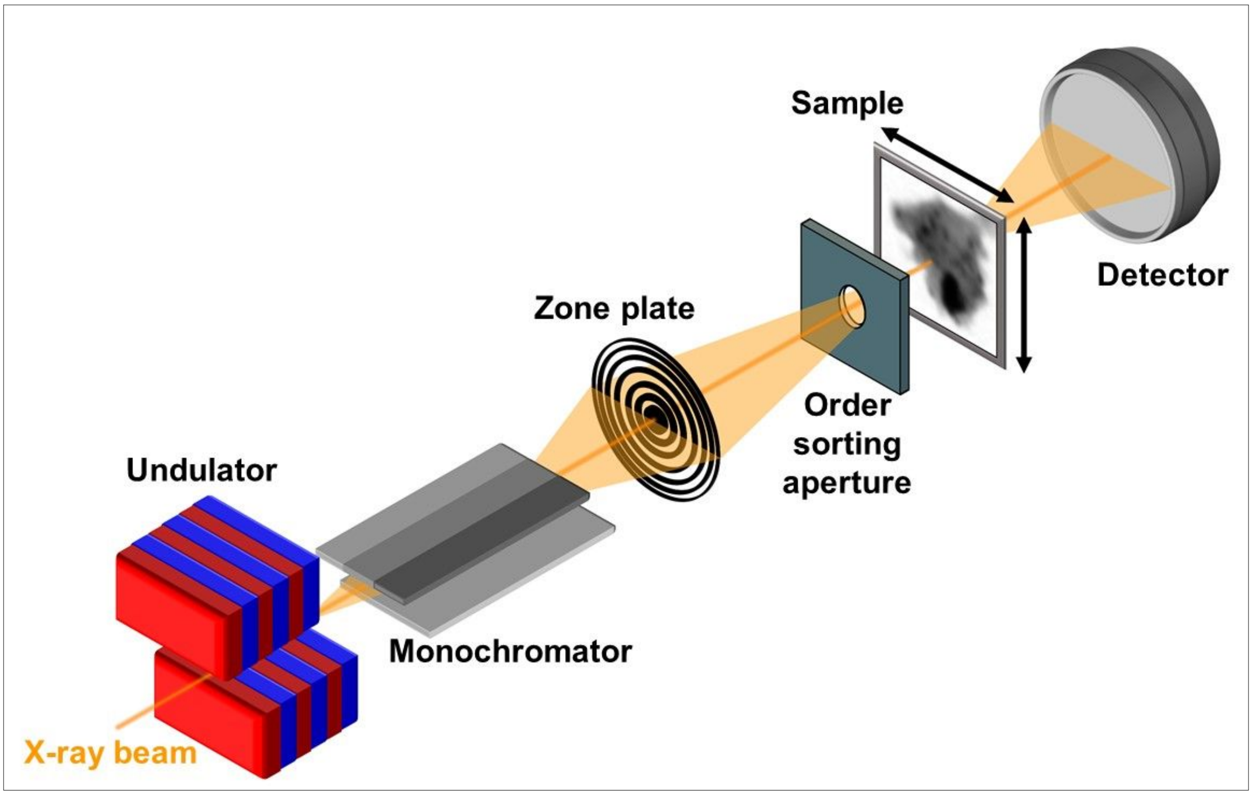

Fig. S21 Schematic showing the scanning transmission x-ray microscope used for STXM measurements.

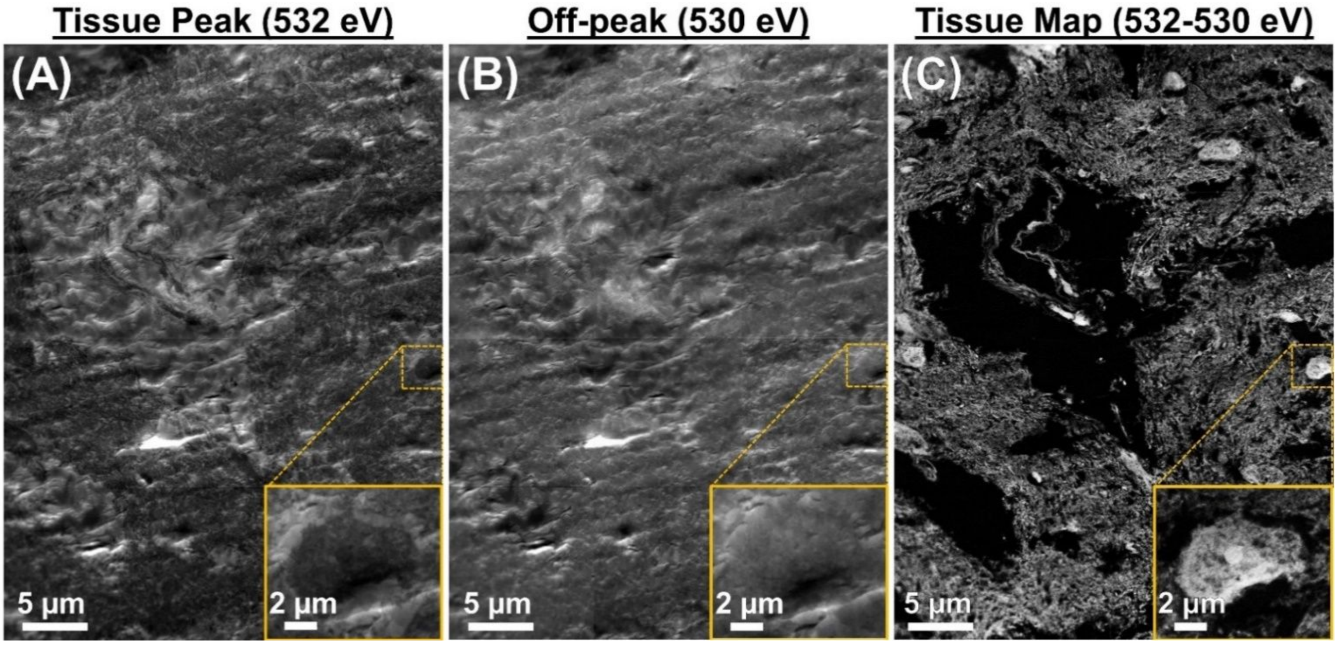

Fig. S22 The methodology of STXM speciation mapping for visualizing tissue structure. Measurements were performed at the oxygen *K*-edge on a 500 nm thick putamen tissue section from a confirmed Alzheimer's disease (AD) case. **(A)** 532 eV x-ray image, corresponding to the peak absorption energy for COO/COOH/$CONH_2$ groups at the oxygen *K*-edge (3). **(B)** Off-peak 530 eV x-ray image. **(C)** Speciation map showing tissue structure created by subtracting the off-peak image (B) from the tissue peak image (A). Cellular and neuropil components of the tissue are clearly identifiable in the tissue map, that were hidden in the single energy images. Inset (yellow box) shows a high resolution images taken over a cellular body within the tissue section, where subcellular features are apparent. An analogous process of tissue mapping can be performed on thinner (<200 nm thickness) tissue sections at the carbon *K*-edge, using the $1s \rightarrow \pi^*$ transition for the C=O bond of amide groups at *ca*. 288.3 eV (see main text Fig. 2) (4).

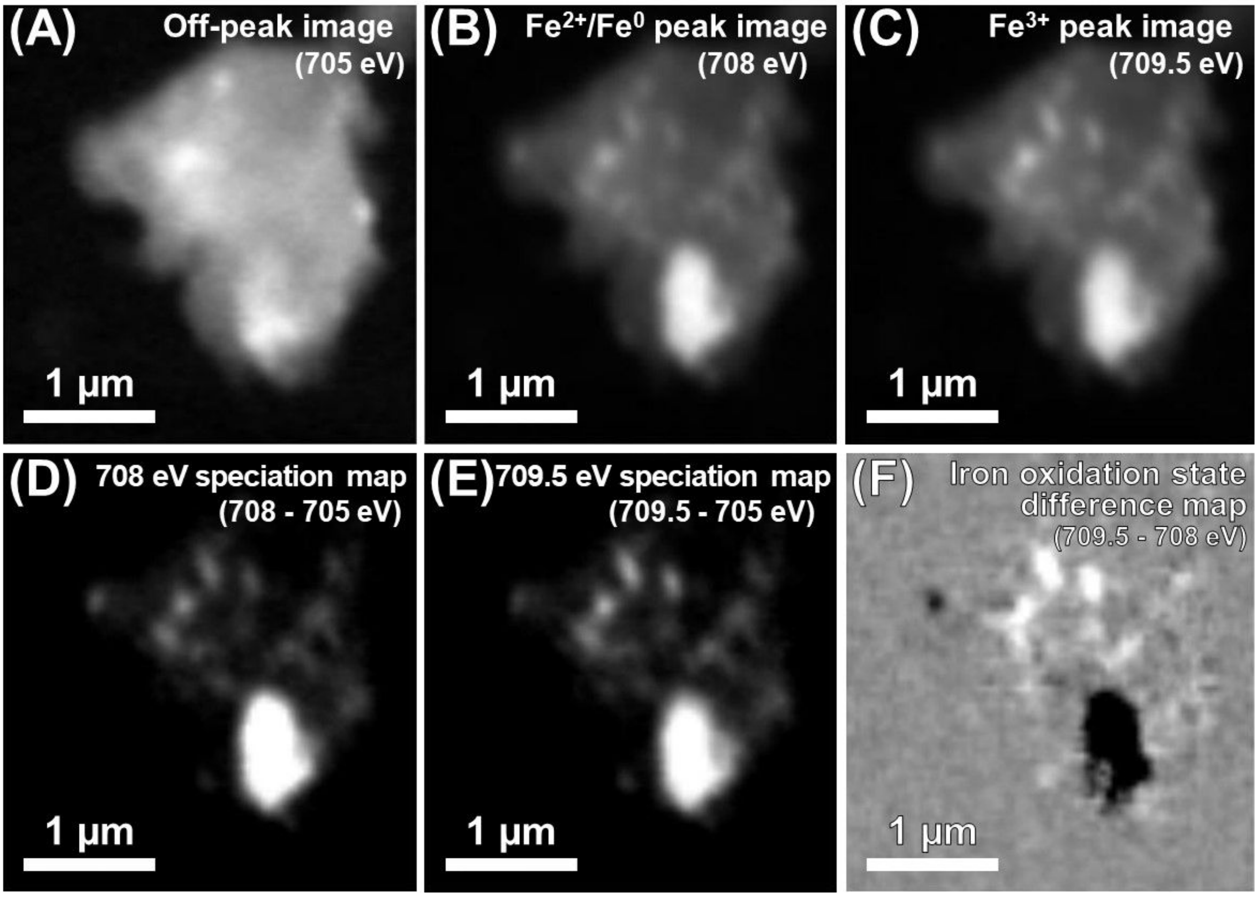

Fig. S23 The methodology of STXM metal speciation mapping. Measurements were performed at the iron L_3-edge on the amyloid plaque shown in Figs. 5-6 of the main text, located within an AD amygdala tissue section. **(A)** Off peak, 705 eV x-ray image. Iron L_3-edge **(B)** Fe^{2+}/Fe^{0} (i.e. Fe^{2+} and/or Fe^{0}) and **(C)** Fe^{3+} peak images. Iron L_3-edge **(D)** 708 eV map and **(E)** 709.5 eV map created by subtracting the off peak image (A), from Fe^{2+}/Fe^{0} (B) and Fe^{3+} (C) peak images respectively. **(F)** Iron oxidation state difference map, created by subtracting (B) from (C). Strongly absorbing oxidized iron (Fe^{3+}) is shown as light contrast, and chemically reduced iron (Fe^{2+} and/or Fe^{0}) is shown as dark contrast. Nanoscale variation in iron oxidation state can be observed.

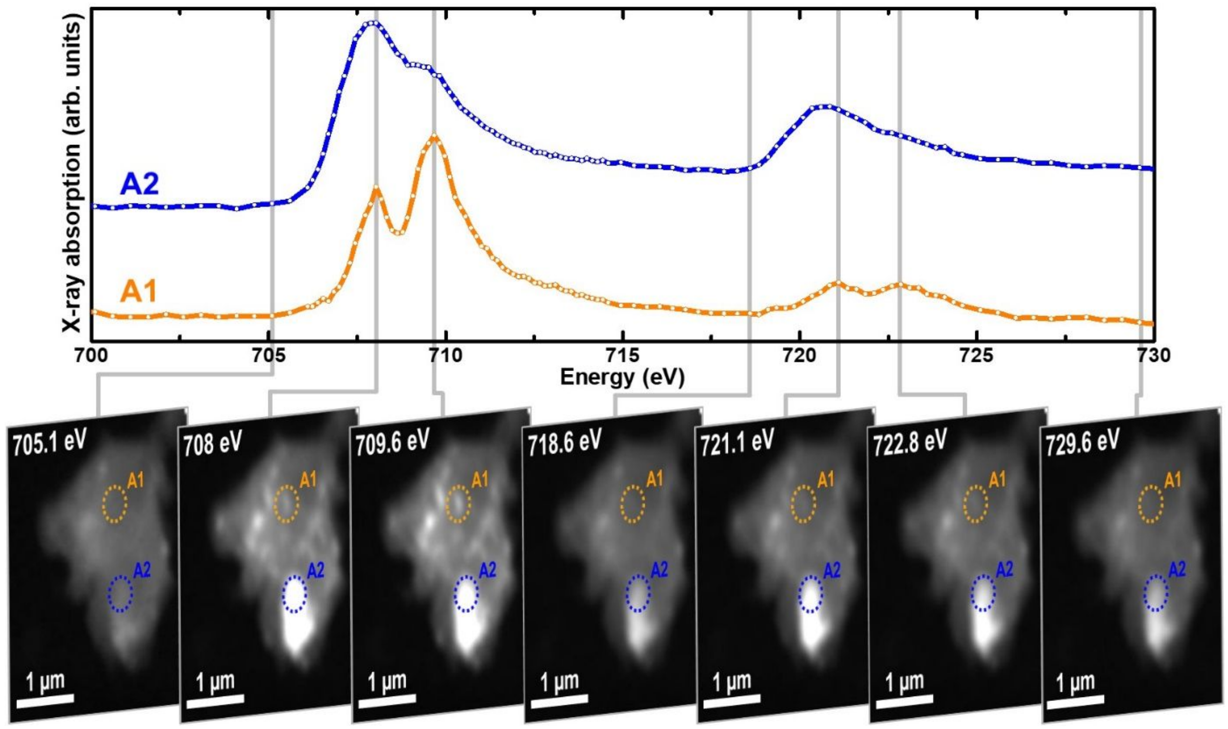

Fig. S24 The methodology for obtaining x-ray absorption spectra using STXM. Measurements were performed over the iron $L_{2,3}$-edge on the amyloid plaque shown in Figs. 5-6 of the main text. In this example, each point of the orange and blue spectra (top panel) correspond to the level of x-ray absorption from the highlighted areas (orange and blue circles) in x-ray images (bottom panels) collected at the specified energy shown by grey lines on the y-axis of the spectra plot. A dramatic variation in oxidation state can be seen in the x-ray absorption spectra from the differing regions within the same plaque.

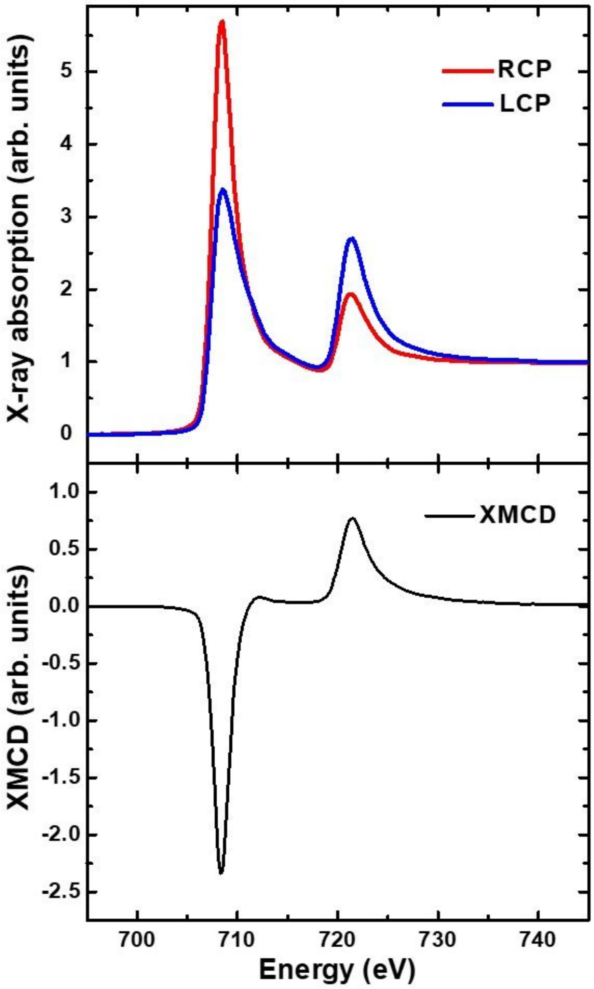

Fig. S25 Iron $L_{2,3}$-edge XMCD measurements of a thin ferromagnetic Fe^0 film standard. Top panel: Left and Right Circularly Polarized (LCP and RCP) x-ray ray absorption spectra. Bottom panel: The corresponding XMCD spectra created by subtracting the RCP spectra from the LCP spectra. The Fe^0 film was prepared and measured under vacuum to prevent oxidation. X-ray absorption spectroscopy measurements were performed under a 0.6 T field at the Synchrotron Radiation Source (Daresbury Laboratory, Cheshire, UK) Beamline 1.1 (5).

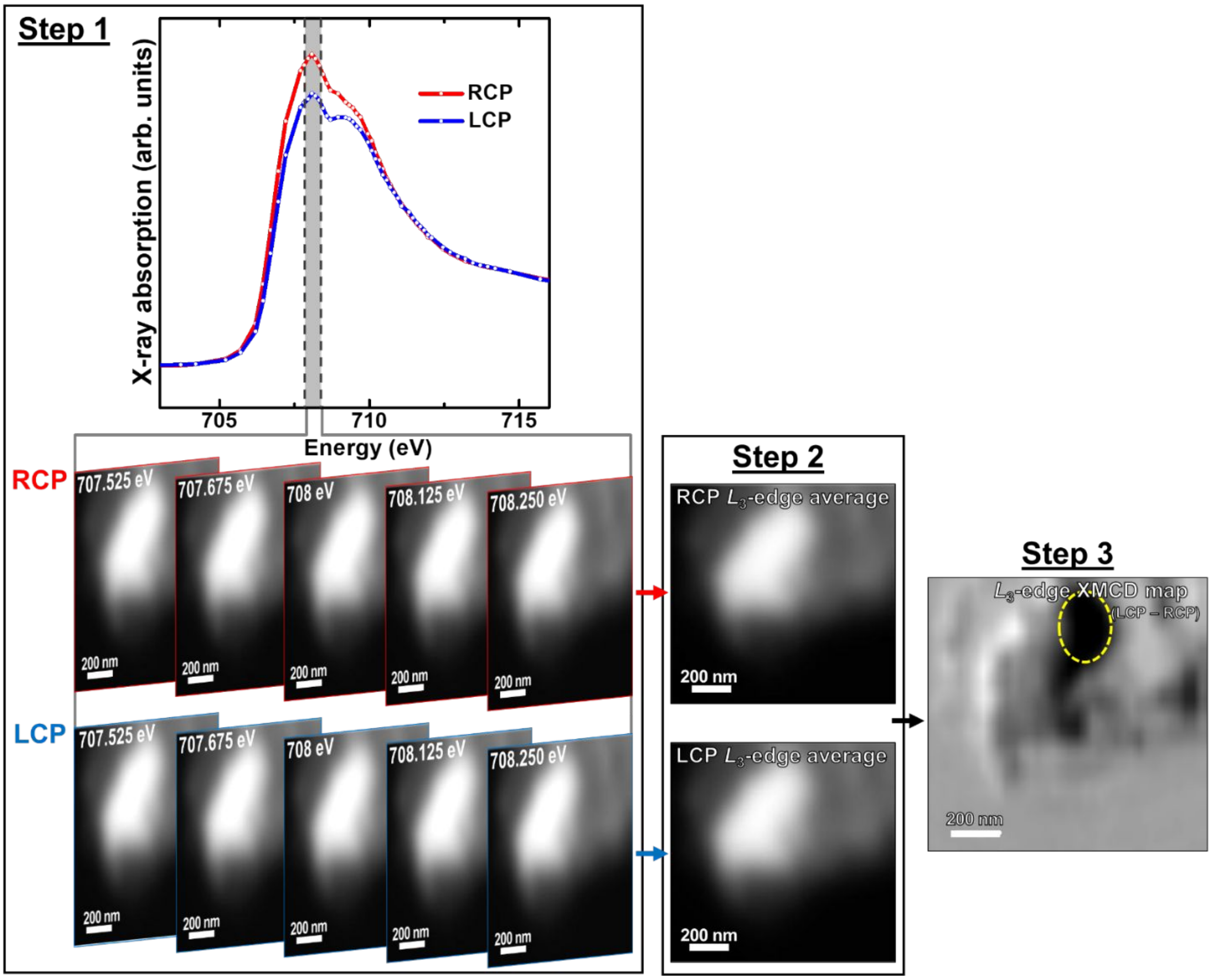

Fig. S26 The methodology for generating iron L_3-edge XMCD speciation maps using STXM. Measurements were performed on the amyloid plaque region shown in Fig. 6 of the main text. **Step 1**: a series of images from an iron region of interest are collected over a desired energy range using RCP and LCP x-rays. Displayed x-ray absorption spectra are collected from the area highlighted in yellow in step 3. **Step 2:** X-ray images from five energy positions spanning an iron x-ray absorption feature of interest (in this case the principal Fe^{2+}/Fe^{0} L_3-edge feature, highlighted by the grey shading in the top x-ray absorption spectra panel) are averaged for each polarization. **Step 3:** The averaged RCP image is subtracted from the averaged LCP image yielding an XMCD difference map where areas of bright or dark contrast indicating significant dichroism effects.

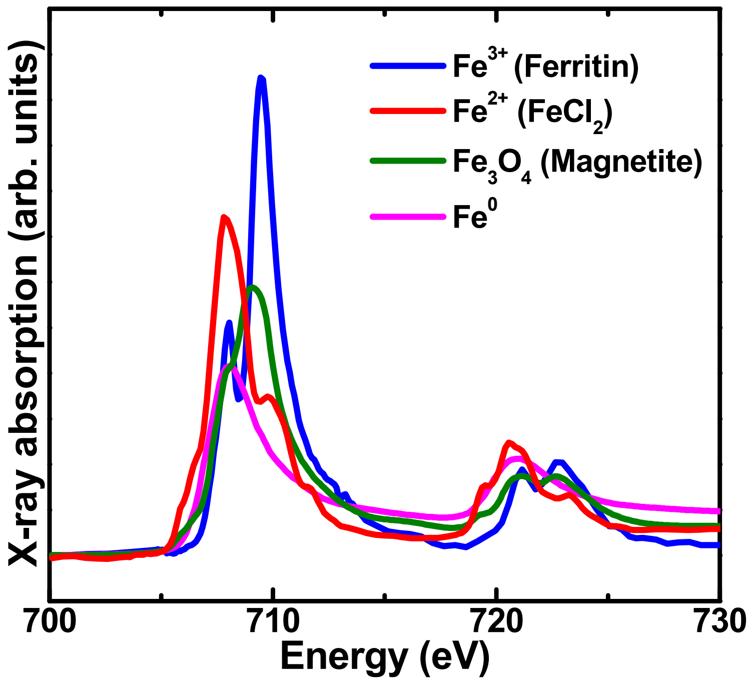

Fig. S27 Scaled iron $L_{2,3}$-edge x-ray absorption reference spectra used to fit experimental iron $L_{2,3}$-edge spectra.

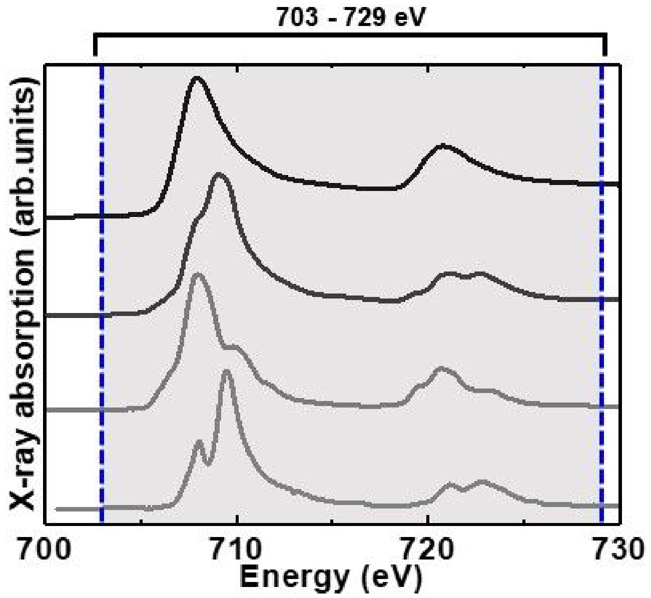

Fig. S28 The energy range used for fitting of the experimental iron $L_{2,3}$-edge x-ray absorption spectra.

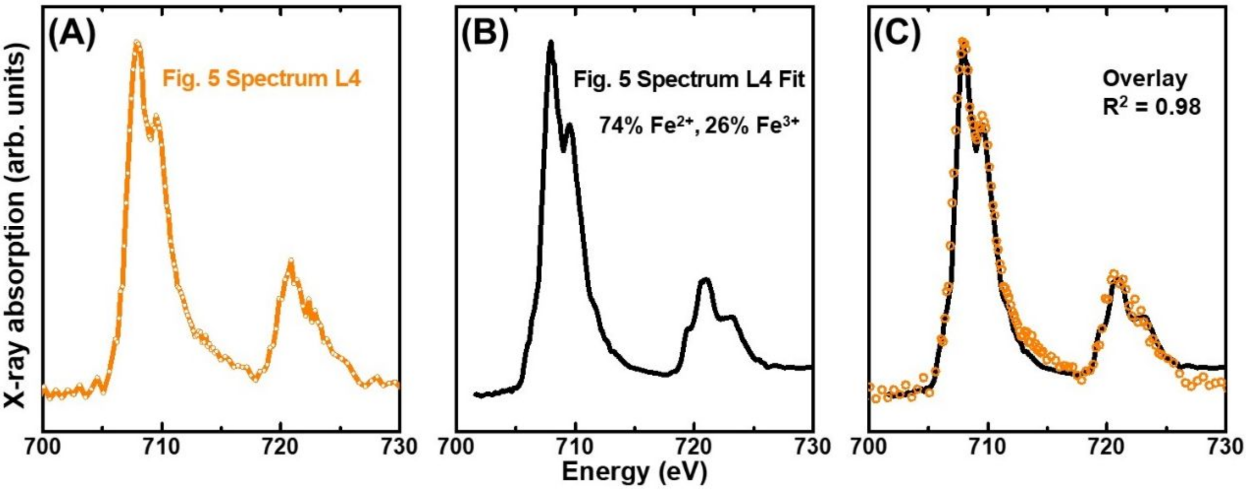

Fig. S29 An overlay plot created from the fitting of experimental iron $L_{2,3}$-edge x-ray absorption spectrum L4 from Fig. 5 of the main text. **(A)** Experimental spectrum, **(B)** corresponding fit, **(C)** overlay showing experimental data (coloured circles) and fit (black line). An R^2 value describing the strength of correlation between the fit and the experimental spectrum is also provided.

Table S1. Fitting values for the displayed experimental iron $L_{2,3}$-edge spectra.

Spectrum	Calculated Fit	R^2 Value
Figure 5 L1	68% Fe^{3+}, 32% Fe^{2+}	0.96
Figure 5 L2	56% Fe^{2+}, 44% Fe^{3+}	0.95
Figure 5 L3	58% Fe^{2+}, 42% Fe^{3+}	0.96
Figure 5 L4	74% Fe^{2+}, 26% Fe^{3+}	0.98
Figure 5 L5	88% Fe^0, 8% Fe^{3+}, 4% Fe^{2+}	0.99
Figure 7 K1	39% Fe^{2+}, 37% Fe^{3+}, 24% Fe_3O_4	0.94
Figure 7 K2	37% Fe^{2+}, 33% Fe_3O_4, 30% Fe^{3+}	0.92
Figure 7 K3	39% Fe^{2+}, 31% Fe_3O_4, 30% Fe^{3+}	0.93
Figure 7 K4	58% Fe^{2+}, 42% Fe^{3+}	0.95
Figure 7 K5	56% Fe^{2+}, 35% Fe^{3+}, 9% Fe_3O_4	0.91
Figure 7 K6	64% Fe^{2+}, 36% Fe^{3+}	0.94
Figure S16	70% Fe^{3+}, 30% Fe^{2+}	0.96
Figure S17 K1	87% Fe^{3+}, 13% Fe^{2+}	0.94
Figure S17 K2	77% Fe^{3+}, 23% Fe^{2+}	0.98
Figure S17 K3	68% Fe^{3+}, 32% Fe^{2+}	0.98
Figure S18	60% Fe^{3+}, 24% Fe^{2+}, 16% Fe_3O_4	0.84
Figure S20 J1	65% Fe^{3+}, 35% Fe^{2+}	0.88
Figure S20 J2	60% Fe^{3+}, 40% Fe_3O_4	0.97
Figure S20 J3	54% Fe^{3+}, 46% Fe_3O_4	0.97
Figure S20 J4	31% Fe^0, 28% Fe^{3+}, 27%$^{2+}$, 14% Fe_3O_4	0.94
Figure S22	59% Fe^{3+}, 32% Fe^0, 9% Fe^{2+}	0.95
Figure S23 K1	100% Fe^{3+}	0.92
Figure S23 K2	94% Fe^{3+}, 6% Fe^{2+}	0.98
Figure S24	56% Fe_3O_4, 37% Fe^{3+}, 7% Fe^{2+}	0.95
Figure S26 F1	87% Fe^{3+}, 13% Fe^{2+}	0.97
Figure S26 F2	70% Fe^{3+}, 30% Fe^{2+}	0.98
Figure S26 F3	62% Fe^{2+}, 38% Fe^{3+}	0.95
Figure S26 F4	65% Fe^{2+}, 35% Fe^{3+}	0.98
Figure S27 H1	69% Fe^{3+}, 31% Fe^{2+}	0.94
Figure S27 H2	65% Fe^{3+}, 35% Fe^{2+}	0.92

References

1. Everett J, Collingwood JF, Tjendana-Tjhin V, Brooks J, Lermyte F, Plascencia-Villa G, et al. Nanoscale synchrotron X-ray speciation of iron and calcium compounds in amyloid plaque cores from Alzheimer's disease subjects. *Nanoscale*. 2018; **10**:11782-96

2. Everett J, Tjhin VT, Brooks J, Lermyte F, Hands-Portman I, Dobson J, et al. Nanoscale Examination of Biological Tissues Using X-ray Spectromicroscopy. *Microscopy and Microanalysis*. 2018; **24**(S2):490-1.

3. Frati F, Hunault MOJY, de Groot FMF. Oxygen K-edge X-ray Absorption Spectra. *Chemical Reviews*. 2020; **120**(9):4056-110.

4. Stewart-Ornstein J, Hitchcock AP, Hernández Cruz D, Henklein P, Overhage J, Hilpert K, et al. Using Intrinsic X-ray Absorption Spectral Differences To Identify and Map Peptides and Proteins. *The Journal of Physical Chemistry B*. 2007; **111**(26):7691-9.

5. Telling ND, van der Laan G, Georgieva MT, Farley NRS. Facility for combined in situ magnetron sputtering and soft x-ray magnetic circular dichroism. *Review of Scientific Instruments*. 2006;77(7):073903.
